# Wavelet Representation of the Corneal Pulse for Detecting Ocular Dicrotism

**DOI:** 10.1371/journal.pone.0124721

**Published:** 2015-04-23

**Authors:** Tomasz Melcer, Monika E. Danielewska, D. Robert Iskander

**Affiliations:** Department of Biomedical Engineering, Wroclaw University of Technology, Wroclaw, Poland; Casey Eye Institute, UNITED STATES

## Abstract

**Purpose:**

To develop a reliable and powerful method for detecting the ocular dicrotism from non-invasively acquired signals of corneal pulse without the knowledge of the underlying cardiopulmonary information present in signals of ocular blood pulse and the electrical heart activity.

**Methods:**

Retrospective data from a study on glaucomatous and age-related changes in corneal pulsation [PLOS ONE 9(7),(2014):e102814] involving 261 subjects was used. Continuous wavelet representation of the signal derivative of the corneal pulse was considered with a complex Gaussian derivative function chosen as mother wavelet. Gray-level Co-occurrence Matrix has been applied to the image (heat-maps) of CWT to yield a set of parameters that can be used to devise the ocular dicrotic pulse detection schemes based on the Conditional Inference Tree and the Random Forest models. The detection scheme was first tested on synthetic signals resembling those of a dicrotic and a non-dicrotic ocular pulse before being used on all 261 real recordings.

**Results:**

A detection scheme based on a single feature of the Continuous Wavelet Transform of the corneal pulse signal resulted in a low detection rate. Conglomeration of a set of features based on measures of texture (homogeneity, correlation, energy, and contrast) resulted in a high detection rate reaching 93%.

**Conclusion:**

It is possible to reliably detect a dicrotic ocular pulse from the signals of corneal pulsation without the need of acquiring additional signals related to heart activity, which was the previous state-of-the-art. The proposed scheme can be applied to other non-stationary biomedical signals related to ocular dynamics.

## Introduction

Ocular dicrotism, manifested as a double-peak shaped feature in corneal pulsation [1–3], is a newly observed phenomenon [4, 5]. Using an innovative noninvasive and noncontact ultrasonic technique [6, 7], ocular dicrotic pulse (ODP) has been observed in a substantial proportion of older subjects (about 70%) but was not evident in the population of younger participants of the study (≤ 35 y.o.) [4]. Further investigation, in which healthy subjects as well as glaucoma patients participated, revealed higher incidence of ODP in two groups of patients: those with open angle glaucoma and those with primary angle-closure glaucoma [5]. Additionally, it was found that the ODP incidence increases with age and that it increases more rapidly in glaucoma patients than in healthy subjects. Both studies concluded that ODP is the natural sign of aging and may have hemodynamic etiology. Automatizing the ODP detection process is of particular importance in large longitudinal studies, involving hundreds of subjects and multiple measurements of the corneal pulse (CP) signal [5].

The detection of ODP is not a trivial task as the corneal pulsation signal measured in terms of CP reveals some non-stationarity. From a mathematical point of view, the CP signal is a non-stationary time series that exhibits a range of behaviors that makes analysis difficult. A raw CP signal contains low-frequency components (ranged 0.2–0.3 Hz) related to respiration, frequencies corresponding to the fundamental frequency of heartbeat (around 1 Hz) and its subsequent frequency harmonics, higher-frequency components related to the device measurement noise, time-localized artifacts of blinking and head movements, as well as general variability in shape and length of subsequent cycles. So far, to eliminate the unwanted signal components from the ocular pulse, a range of filtering techniques have been applied [4].

Previously suggested algorithm for detecting ODP, based on Dynamic Time Warping (DTW) signal averaging in the time domain [8, 9], required synchronous measurement of heart activity signals (e.g., blood pulse signal or the ECG signal) [4]. This was necessary because the CP signal does not exhibit clearly defined peaks such as those encountered in the blood pulse or ECG signals (e.g. R-R’ peaks). Additionally, cases were encountered in which the DTW algorithm misrepresented signal features and had to be manually corrected. In the frequency domain, spectral analysis, based on energy estimates, seem to be adequate for signals with slowly varying frequency contents [10, 11]. Nevertheless, recent attempt to detect ODP from CP signals alone showed the task to be more intricate than expected. In particular, it has been recently found that in order to separate the class of ODP signals from that of non-ODP signals, spectral analysis of both CP and BPL signals (in terms of coherence) had to be considered.

Our aim is to ascertain whether the content of CP signal alone carries enough information for ODP detection to be performed reliably and whether information from the auxiliary physiological signals such as those of blood pulse or electrical heart activity is necessary to perform this detection task effectively. An obvious alternative to previously considered time and frequency domain techniques are methods dedicated to non-stationary signal analysis such as wavelet transform.

## Materials and Methods

We have used retrospective data from a study reported in [5]. The study was approved by the Ethics Committee of Wroclaw Medical University (decision No KB 503/2011) and adhered to the Tenets of the Declaration of Helsinki. 261 subjects participated in that study including 191 patients of the Glaucoma Clinic (Wroclaw Medical University) and a control group (CG) of 70 healthy individuals. The patients were classified into two groups including those with an ocular dicrotic pulse (189 subjects) and those without it (72 subjects). In the study, corneal pulse (CP) signal was measured using a non-contact method based on an ultrasonic distance sensor in air [7]. Synchronically, blood pulse (BPL) signal, acquired from the right earlobe with a pulse oximeter, and the ECG signal, registered in a standard three-lead system of Eindhoven triangle, were also recorded. Only CP recordings were subsequently used because the purpose of this study was to detect the ODP from the CP signal alone. Each recording was a 10 seconds long signal sampled at 400 Hz. All patient records and information was anonymized and de-identified prior to analysis.

The method proposed in this paper consists of applying Continuous Wavelet Transform to a raw CP signal. Then, in order to determine the feasibility of recognizing ODP from a raw CP signal alone, we extract texture features from the resulting wavelet image using Gray-level Co-occurrence Matrix (GLCM). In the final step, we apply statistical methods to the extracted features in order to find a reliable and powerful detector of ODP.

### Continuous Wavelet Transform

Wavelet transform [12] is a method of processing a digital signal in order to reveal time-frequency features of the signal. The wavelet transform has found its use in many biomedical applications [13] including analysis of biomedical signals related to the cardiopulmonary system [14–16]. Commonly used wavelet transforms belong to two classes: a Discrete Wavelet Transform, predominantly used in compression schemes [17] due to its compact representation, and Continuous Wavelet Transform (CWT), which features an ability to localize and match predefined shapes in a non-stationary noisy signal.

For the purpose of the ODP detection, a wavelet is defined as any complex-valued function that has compact support (i.e., it is zero-valued outside a finite interval) and integrates to zero. A certain class of wavelets defined by a representant called a mother wavelet is chosen. The mother wavelet defines the shape of signal features being matched. A single wavelet of scale *s* is then constructed by sampling and *L* ^1^-normalizing the mother wavelet so that support of the resulting signal is exactly of length *s* samples and centered at *t* = 0. Each scale highlights features of certain size or frequency, but the correspondence varies depending on the chosen mother wavelet.

To perform a Continuous Wavelet Transform, we compute a set of convolutions of the signal with wavelets of scales corresponding to frequencies of interest. It is customary to perform the convolutions for scales from an equidistant grid of values on a linear or logarithmic scale from a certain interval; this procedure is similar in purpose to a band-pass filter, as the frequencies corresponding to scales from outside the interval will not be represented in the output. Therefore, explicitly filtering out low and high frequencies before applying the wavelet transform is not necessary.

This computation takes *O*(*w*â€‰*n*log*n*) time, for *w* being number of different scales and *n* being length of the input signal. This is because we compute *w* convolutions, each convolution can be computed using a fast convolution algorithm based on Fast Fourier Transform in *O*(*n*log*n*) time.

Visualization is then prepared as a set of four heat-maps where each row corresponds to the real part, imaginary part, modulus or phase of the result of the convolution at a certain scale, with scales growing along the Y axis. To avoid misinterpretation of values at the edges of the heat-maps, we chose to blank out the parts of heat-maps where the non-zero support interval of a wavelet fell outside the observation interval. This action results in a trapezoid shape of the final heat-maps.

As the wavelet transform is a result of a set of convolutions, it is a linear time-invariant filter. The output of the filter does not change after adding a constant to the input signal; scaling input by a factor scales the output by the same factor. Therefore the final visualization is not affected by either translation or scaling of the input signal, which means that preprocessing steps that affect the scale of the input values are not necessary.

The effect of an independent additive noise in the input signal on the output values is equal to an additive noise applied to the output values, with standard deviation equal to the standard deviation of the input noise multiplied by the *L* ^2^ norm of the wavelet signal. As the wavelet is already an *L* ^1^-normalized signal, this means that the output noise decreases with increasing scale and even for small scales it is always smaller in magnitude compared to the input.

### Choice of mother wavelet and scales

**Fig 1:**
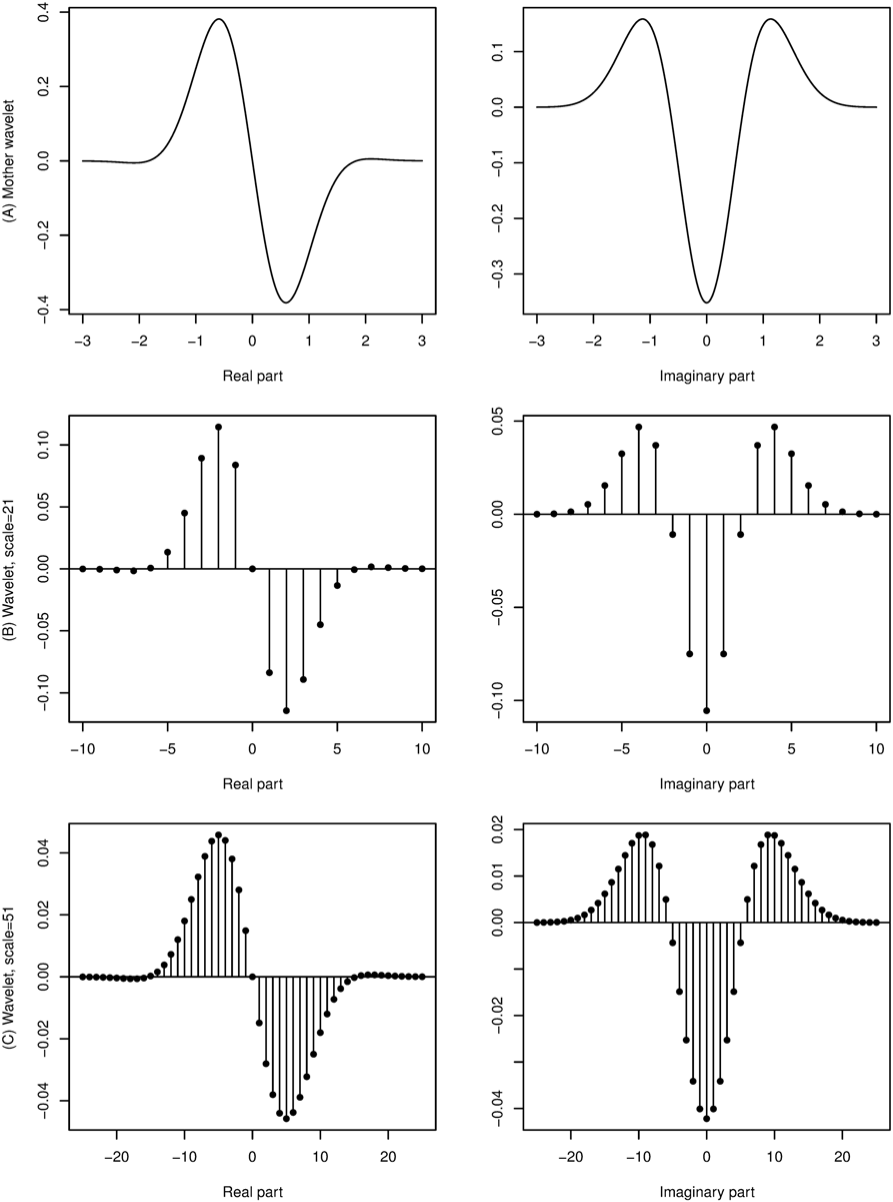
Example of a mother wavelet function. From top: (A) complex Gaussian derivative mother wavelet *f*(*t*) = (−2*x*−*i*)exp(−*x* ^2^−*ix*), (B) example wavelet of scale *s* = 21, (C) example wavelet of scale *s* = 51.

It has been noticed [18] that matching features on the derivative of the raw signal instead of performing the convolution on the raw signal itself has better localization properties. However, to match raw signal features on the derivative of the signal, the wavelet must have a shape that corresponds to a derivative of the expected raw signal feature. The measured CP signal is an approximation of a continuous physical process and the features of interest have a shape of a wide peak. Because of that we have decided to use an *L* ^1^-normalized derivative of a complex Gaussian function *f*(*t*) = exp(−*x* ^2^+*ix*), for *t* ∈ [−5,5] as a mother wavelet (see Fig 1).

For presentation and visual evaluation purposes CWT was computed for scales from interval [10, 3500], that is, for scales covering almost all technically possible scale values for a signal of length 4000. After visual evaluation of CWT heat-maps computed for a small subset of registered signals, we constrained the scales to an interval [200, 1000] before applying statistical learning methods. This was done to focus on the parts of CWT images that differ the most between various recordings and to speed up computations.

### Extracting image features

Statistical classifiers require features in form of numerical statistics to work. One of the methods to extract numerical statistics from CWT is to treat the CWT image as a texture and compute various image parameters using Gray-level Co-occurrence Matrix method (GLCM) [19]. Specifically, the CWT image is firstly quantized into N levels. An N-by-N matrix (*A*)_*ij*_ is computed so that *a*_*ij*_ contains a number of horizontally neighboring pixel pairs of the input image where the first pixel has level *i*, and the second pixel has level *j*. Depending on the use case, the pixels might be separated by a specified horizontal distance *L* expressed in number of pixels, ie. pixels do not have to be direct neighbors. The matrix is then normalized and statistics such as homogeneity, energy, contrast and correlation are computed.

This procedure was applied to all four heat-maps representing the real and imaginary part, modulus and phase of the CWT. As a result, we collected 16 numerical features. We found *N* = 8 levels to be sufficient for this work. As CWT with a continuous wavelet function results in smooth image, computing GLCM for direct neighbors would put a lot of probability mass in the normalized matrix onto the diagonal. To lessen this effect and increase the range of the computed statistics, we have chosen *L* = 25, corresponding to 0.0625 seconds of delay.

### Numerical simulations

To characterize basic properties of the proposed wavelet transform method in an ideal setting a periodic sinusoidal signal with frequency of 1 Hz and a signal composed of two sinusoidal components of 1 Hz and 2 Hz were used. The second signal components were chosen to imitate an ODP signal. Two additional factors were considered: additive white Gaussian noise at the level of 25 dB corresponding to a typical value of signal to noise ratio (SNR) for biomedical signals and a component with frequency of 0.3 Hz simulating the effect of breathing modulation on registered corneal pulsation signals. All test signal parameters were chosen to match the length, frequency, mean and average amplitude of experimental signals.

To assess utility of the presented wavelet transform in practical scenarios, we have applied the transform to registered CP signal with no evidence of dicrotic shape in its waveform and CP signals with ODP, detected using the previous technique [5] that required the information from the ECG signal as well as bandpass filtering of the signals.

### Detection

We employed two classifier algorithms for the task of detecting ODP: Conditional Inference Tree and the Random Forest. Conditional Inference Tree is a variation of a class of regression tree algorithms, known for statistical soundness and ease of interpretation [20]. Random Forest is a decision tree-based ensemble learning algorithm with natural way of estimating importance of variables and good generalization properties [21]. In both cases we have used existing R implementations of these algorithms, as distributed in packages ctree[22] and randomForest[23].

The statistical learning process was controlled by backward sequential feature selection procedure [24] and the bootstrap 632+ validation method technique [25] in order to avoid overfitting. Both algorithms were implemented by the caret package [26]. The feature selection process was driven by the variable importance ranking built by using an estimate of average loss of entropy criterion computed by Random Forest [27].

## Results

### Synthetic harmonic signals

**Fig 2:**
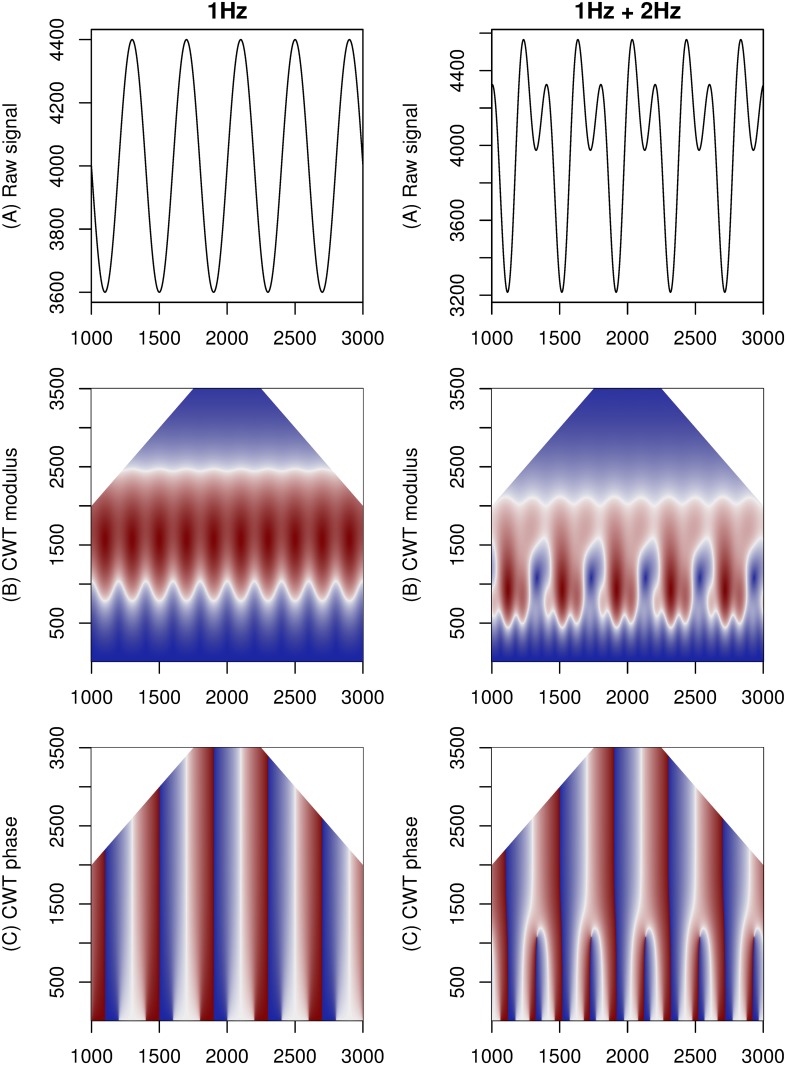
Examples of visualization of 1 Hz (on the left) and composite 1 Hz + 2 Hz harmonic signal (on the right). From top: (A) raw signal, (B) modulus of the wavelet transform, (C) phase of the wavelet transform.

Fig 2 contains visualizations of a 1 Hz harmonic signal and a 1 Hz harmonic signal composed with a 2 Hz harmonic. As evidenced by the images of the CWT, both visualizations contain distinctive patterns that relate to the original signals. The 1 Hz harmonic signal is represented on the modulus heat-map by elliptical areas at scales between 800 and 2500, two for every cycle, each one with a single maximum. It can be observed that locations of these maxima match the locations of maxima and minima in the raw signal; also their scales are constant. The phase heat-map shows rectangles of alternating sign, bounded by positions matching the extrema of the raw signal.

The composite 1 Hz and 2 Hz signal exhibits more complexity in shape of its CWT. A regular repeating pattern consisting of three overlapping elliptic areas on the modulus heat-map follows the periods of the raw signal. The pattern is located at a constant set of scales. The phase heat-map displays a pattern that matches in location the extrema of the raw signal.

The effect of independent 25 dB white Gaussian noise is shown in figure S1 Fig. The wavelet transform is robust to this kind of noise. Compared to the noiseless images, the noisy images have lower contrast, but all shapes are preserved. Moreover, the locations and scales of the shapes have not been changed. The noise itself is the most visible in the lowest part of the heat-map, for scales below 300. Our other experiments show that even for a signal of 5 dB above the noise level the wavelet transform detects locations and scales of extrema with good results, even if the heat-map’s contrast becomes too low for human to inspect the results.

The CP signal as measured by the ultrasonic distance sensors contains a low-frequency modulating pulmonary system component. The effect of a synthetic modulating signal of similar properties (a 0.3 Hz sinusoid signal) is shown on figure S2 Fig. Major properties of the visualization, compared to the previous ones, do not change up to scales of about 800. Above that scale wavelet shapes become increasingly modulated by the new component. This is especially clearly seen on the 1 Hz modulus heat-map, where only the elliptic shapes which fell close to minima or maxima of the modulating signal are still clearly visible. The phase heat-maps do not change below scale 1000.

### Registered real CP signals

**Fig 3:**
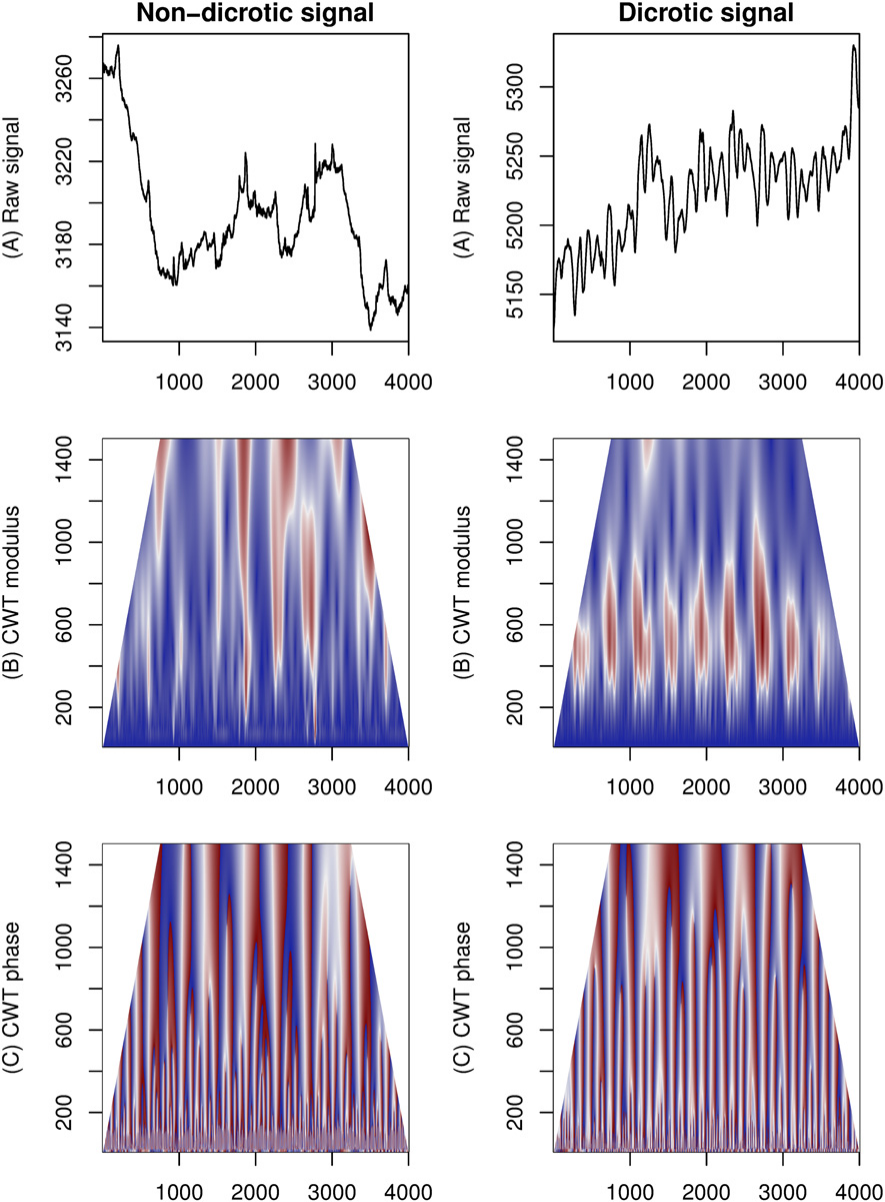
Application of Continuous Wavelet Transform to example non-dicrotic (on the left) and dicrotic (on the right) signals. From top: (A) raw signal, (B) modulus of the wavelet transform, (C) phase of the wavelet transform.

Fig 3 shows the result of application of the Continuous Wavelet Transform to two registered real CP signals. Real signals do not exhibit regularity of the synthetic signals, which results in more complex shapes of the positive regions of the CWT, and their non-uniform placement.

Wavelets at higher scales display lower rate of change compared to wavelets at lower scales. This effect is related to the time-frequency resolution tradeoff typical for multi-scale visualizations, a limitation coming from the Heisenberg uncertainty principle [28]. Large-scale features of the raw signal can be visually matched to large values of the modulus of the CWT at bigger scales. It can also be observed that small features do not influence the parts of CWT that represent wavelets of large scales. No major differences between dicrotic and non-dicrotic signals can be easily observed on the parts of heat-maps that represent scales below 200.

A substantial difference of shape is visible on the modulus heat-map between the non-dicrotic and dicrotic signals. The non-dicrotic signal does not have regular features in the scales visible on the heat-map. The ODP signal has a distinct noisy shape placed in regular intervals. This pattern has been observed in all registered signals that exhibit a clear ODP and in none of the signals with no evidence of ODP. The phase heat-map displays irregular change of complex phase computed in CWT. In the non-dicrotic signal, the change is visibly less regular than in the ODP signal.

### Statistical analysis

An exploratory statistical analysis shows that all signals with a clear ODP, and none of the signals with no evidence of ODP, have a low homogeneity value (≤ 0.428) as computed on the phase heat-map of the CWT. When this criterion is applied to all registered samples (including samples close to the previously established boundary of 3 dB difference in peaks [5]), the accuracy of classification of signals by the homogeneity criterion alone is low and achieves detection level rate of about 55%.

**Fig 4:**
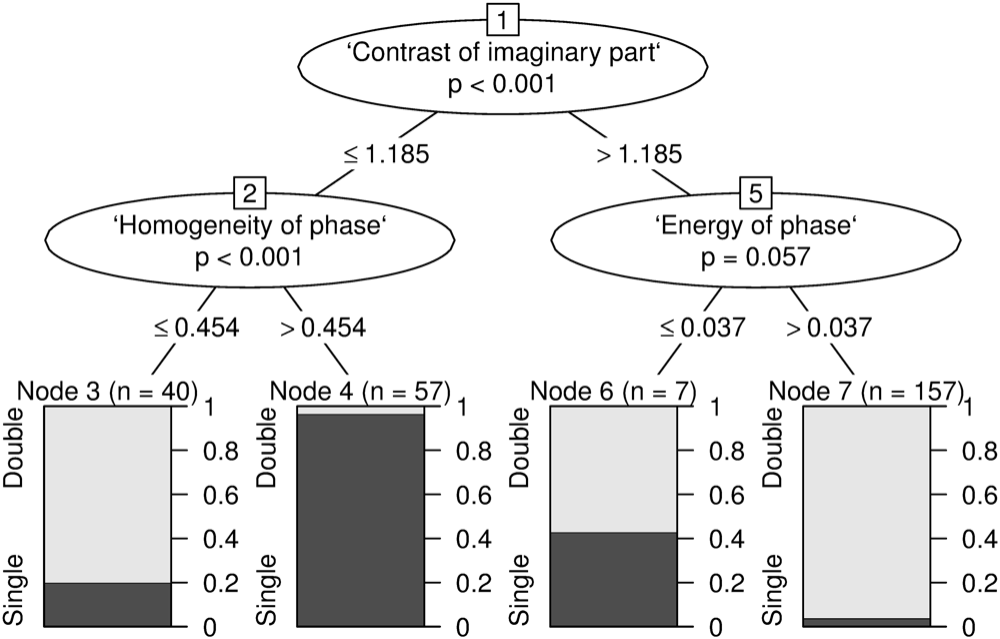
Conditional Inference Tree model of GLCM-based features for detecting ODP.

Further experimentation with a range of multivariate classifiers based on a variety of features extracted from the CWT visualizations proved to result in a much better detection scheme. In an attempt to create an interpretable classifier model, after performing feature selection we trained a Conditional Inference Tree model. The resulting decision tree is presented on Fig 4. Its accuracy is estimated by the bootstrap 632+ method to be 0.90 (standard deviation 0.034). An application of Random Forest method for detection purposes (as opposed to variable importance) produced a model with higher accuracy (0.93, standard deviation 0.031), though this model family is known to be more difficult to interpret. It is worth noting that these accuracy rate results have been calculated in relation to the results obtained in [5].

## Discussion and Conclusions

Dicrotic pulse, also known as the dicrotic arterial wave, is well described in the medical literature [29–31]. On the other hand, ocular dicrotic pulse (ODP) is a newly observed phenomenon related to double peak shape of the corneal pulse (CP) [4, 5]. Of interest is that the ODP could only be observed with non-contact ultrasonic distance sensor but was not evident in measurements of the corneal indentation pulse [32] or the intraocular pressure with a contact dynamic tonometer [33]. Hørven and Nornes [32] were first to realize that the form of the corneal pulse signal reflects pathological alternations in ocular blood supply and may also be related to cluster headache and migraine. Recent results on ocular dicrotism reveal that the shape of the ocular pulse signal changes with age [4] and the considered subject group. In particular, it was shown that the incidence of ocular dicrotism is higher in older glaucomatous eyes than in the corresponding healthy eyes [5].

However, the exact origin of the ocular dicrotism is still unknown. It can be speculated that ODP can be attributed to one or a combination of the following factors: age-related increase in ocular rigidity [34], changes in ocular blood flow [35], choroidal circulation [36], and changes of the biomechanical structure of cornea and sclera [37]. Likewise, the increased incidence of ODP in glaucoma subjects is understood to be the result of more pronounced changes of those factors in glaucomatous eyes than in the control healthy eyes. It is expected that the effects of aging in ocular muscles plays less significant role in the origin of ODP to that of ocular eye globe factors mentioned above.

Corneal pulse signal is highly correlated (i.e., has large coherence function values) with the signal of blood pulse and ECG [11]. Detection of the ODP based on the combined knowledge of CP, blood pulse and ECG signals can reveal important information on the eye’s hemodynamics [38, 39] while the analysis of the CP signal alone may provide more information on biomechanical behavior of cornea to chronically elevated intraocular pressure providing additional clues on the progression of glaucoma [40].

Also, the ability to detect ODP from the CP signal alone has a significant advantage over previously considered methods based on Dynamic Time Warping algorithm [5] and spectral analyses [11]. The latter rely on information on heart rate variability for which synchronous measurement of either blood pulse signal or the ECG is required. This makes the entire data acquisition system more complex and the setup procedure more cumbersome and lengthy.

In general, CP signals are non-stationary and need to be analysed with techniques that extend the spectral analysis to the time and frequency domain. We used Continuous Wavelet Transform but other representations, such as time-frequency distributions (TFD) [41],[11], could have also been used. The representation of the Continuous Wavelet Transform is more difficult to interpret than that of TFD. On the other side, CWT attains more flexibility by allowing mother wavelets to model signal shapes of interest. Moreover, the result of CWT is, assuming a continuous mother wavelet function, also a continuous function, which leads to easier theoretical analysis.

The results of the proposed new ODP detection technique carry two important messages. It is not possible to detect ODP in CP signals alone if we base our detection on a single feature derived from the continuous wavelet representation. However, agglomeration of a set of features results in a reliable scheme that achieves high detection rates reaching 93%. It is important to note that this is possible without acquiring additional signals of blood pulse or electrical heart activity that were necessary to perform this detection task effectively in the past.

From the technical point of view, the proposed methodology has been purposely designed for detecting ocular dicrotism from the corneal pulse signals but in no way is limited to that application. It can be applied to a multitude of biomedical non-stationary signals in which relevant information encoded in the signal’s shape depends on changes or alterations in physiological conditions (e.g., aging, pathologies). In this view, assessment of many non-stationary biomedical signals such as those of encephalography, electrocardiography, electrooculography, and electromyography, to mention just a few, could use the proposed approach that combines wavelet transform with texture analysis and machine learning.

Current knowledge of the ocular dicrotism phenomenon [4, 5] strongly suggests that accurate detection of ODP could support in future early diagnosis of glaucoma, help predicting results of surgical interventions, and support glaucoma management. Also, it is anticipated that this methodology may find its applications in other areas of ophthalmology or even general medicine of systemic diseases, proving that the early attempts of gaining such information from the signals of corneal pulse were not superfluous [1, 2].

## Supporting Information

S1 Fig Examples of visualization of signals with SNR = 25 dB independent white Gaussian noise applied: 1 Hz (on the left) and composite 1 Hz + 2 Hz harmonic signal (on the right). From top: (A) raw signal, (B) modulus of the wavelet transform, (C) phase of the wavelet transform.
(TIFF)

S2 Fig Examples of visualization of signals with breathing-like component: 1 Hz (on the left) and composite 1 Hz + 2 Hz harmonic signal (on the right). From top: (A) raw signal, (B) modulus of the wavelet transform, (C) phase of the wavelet transform.
(TIFF)

S3 Fig A side-by-side comparison of visualizations of modulus of wavelet transform computed for several non-dicrotic and dicrotic signals. (JPG)

S1 Dataset Raw ocular pulse signals (both dicrotic and non-dicrotic) along with precomputed wavelet visualizations and GLCM statistics. (XZ)
