# Supplementary figures and images for: Wavelet Representation of the Corneal Pulse for Detecting Ocular Dicrotism
Source: PLoS One. 2015 Apr 23;10(4):e0124721. doi: 10.1371/journal.pone.0124721 (PMC4408059; doi:10.1371/journal.pone.0124721)

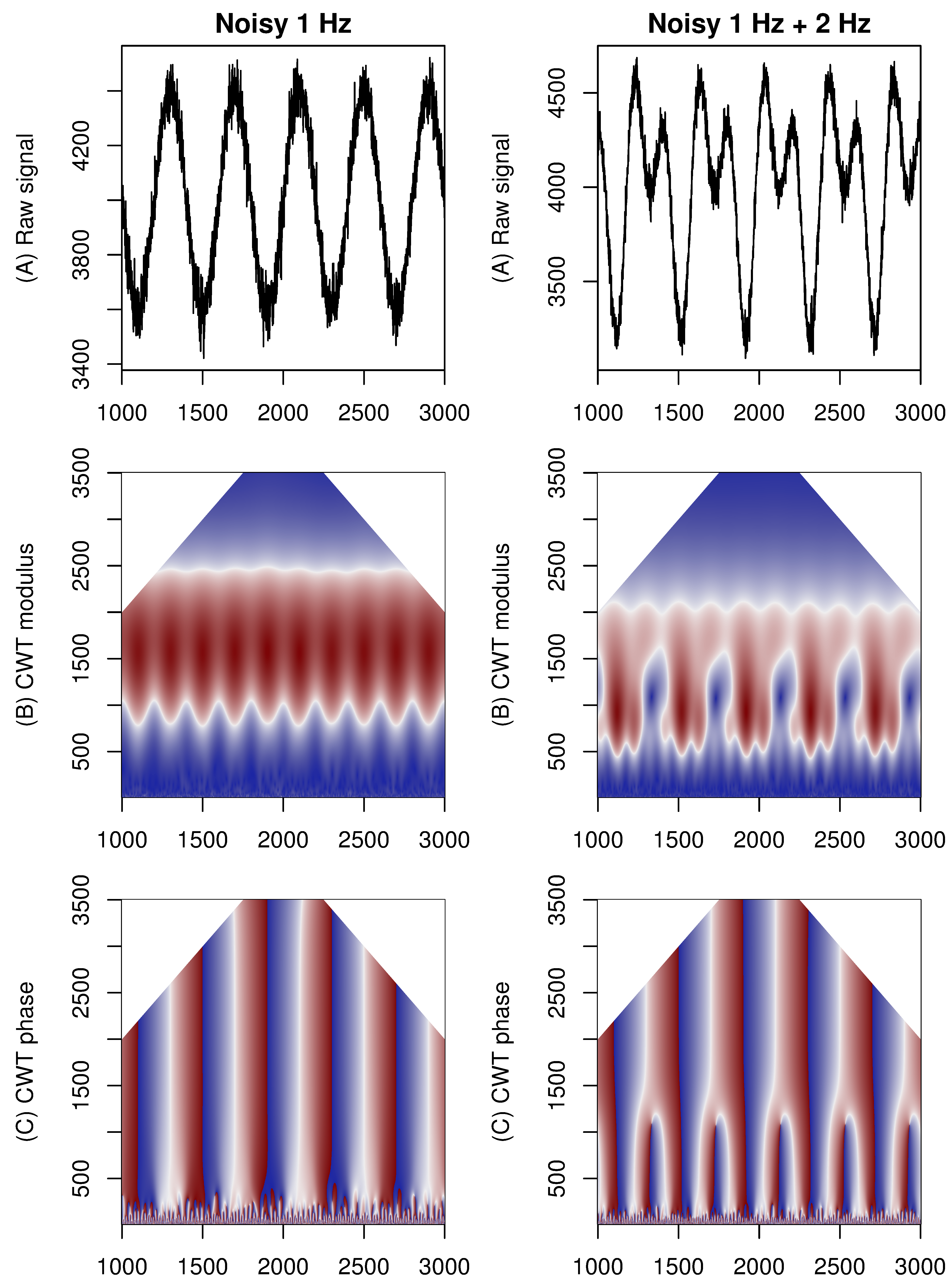

Supplement: S1 Fig — From top: (A) raw signal, (B) modulus of the wavelet transform, (C) phase of the wavelet transform. (TIFF) [file pone.0124721.s001.tiff]

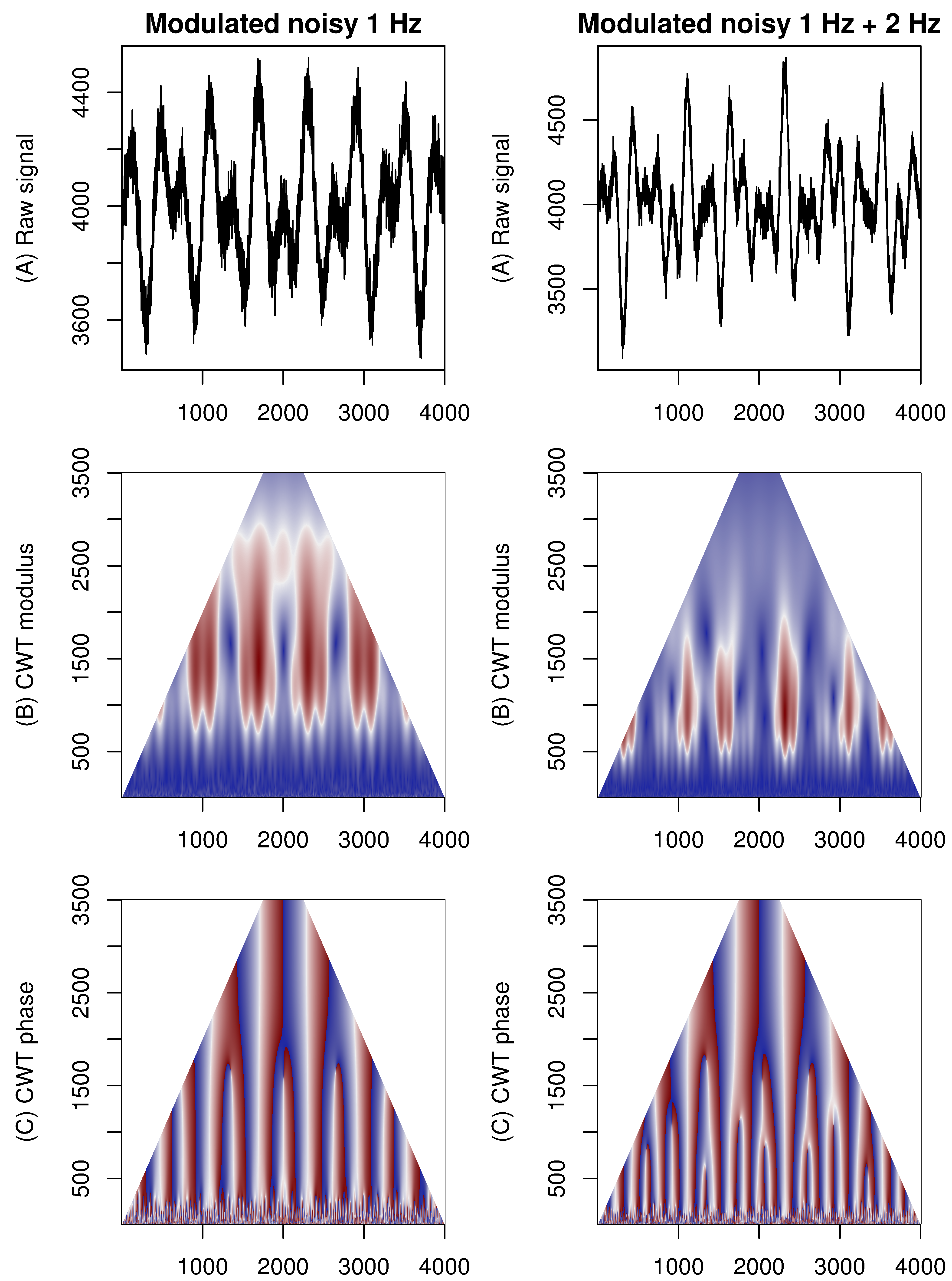

Supplement: S2 Fig — From top: (A) raw signal, (B) modulus of the wavelet transform, (C) phase of the wavelet transform. (TIFF) [file pone.0124721.s002.tiff]

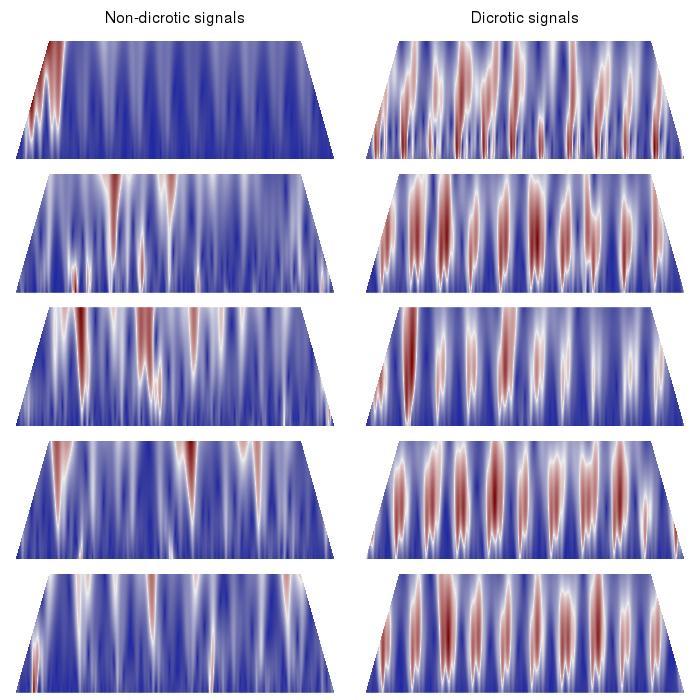

Supplement: S3 Fig — (JPG) [file pone.0124721.s003.jpg]

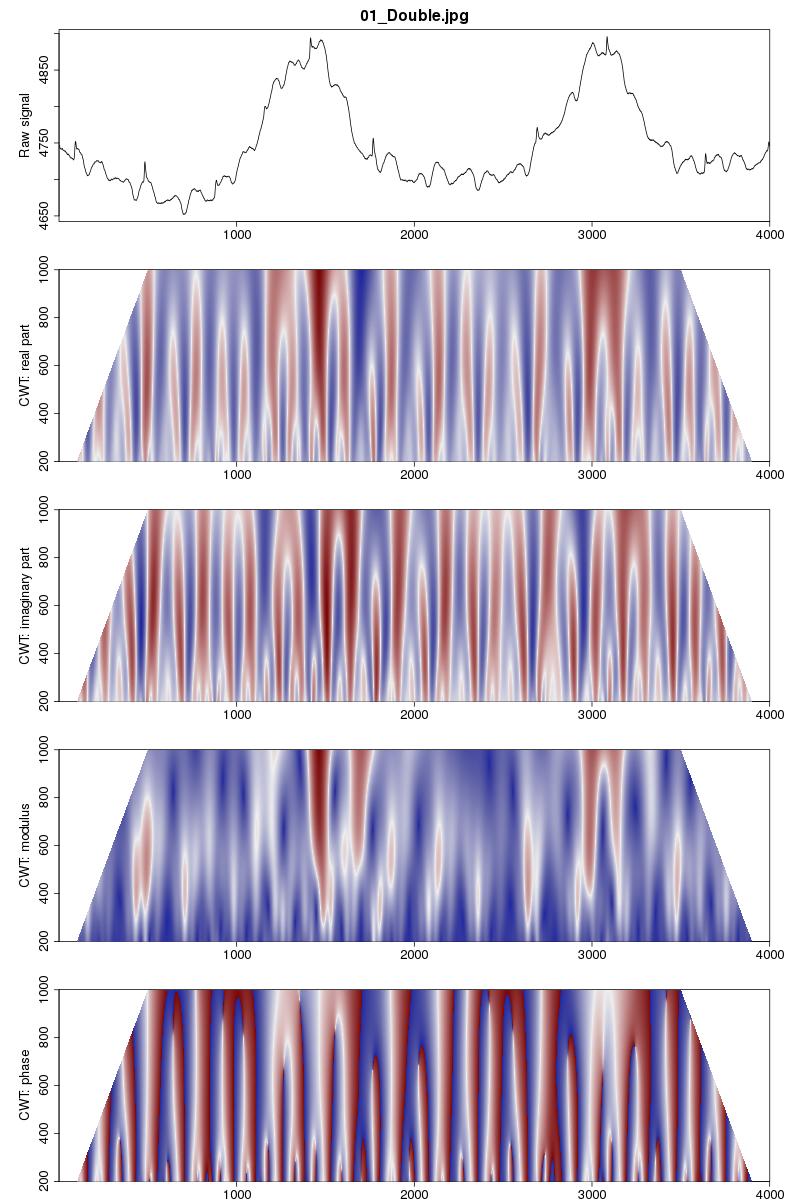

Supplement: S1 Dataset — (XZ) [file pone.0124721.s004.xz › 01_Double.jpg]

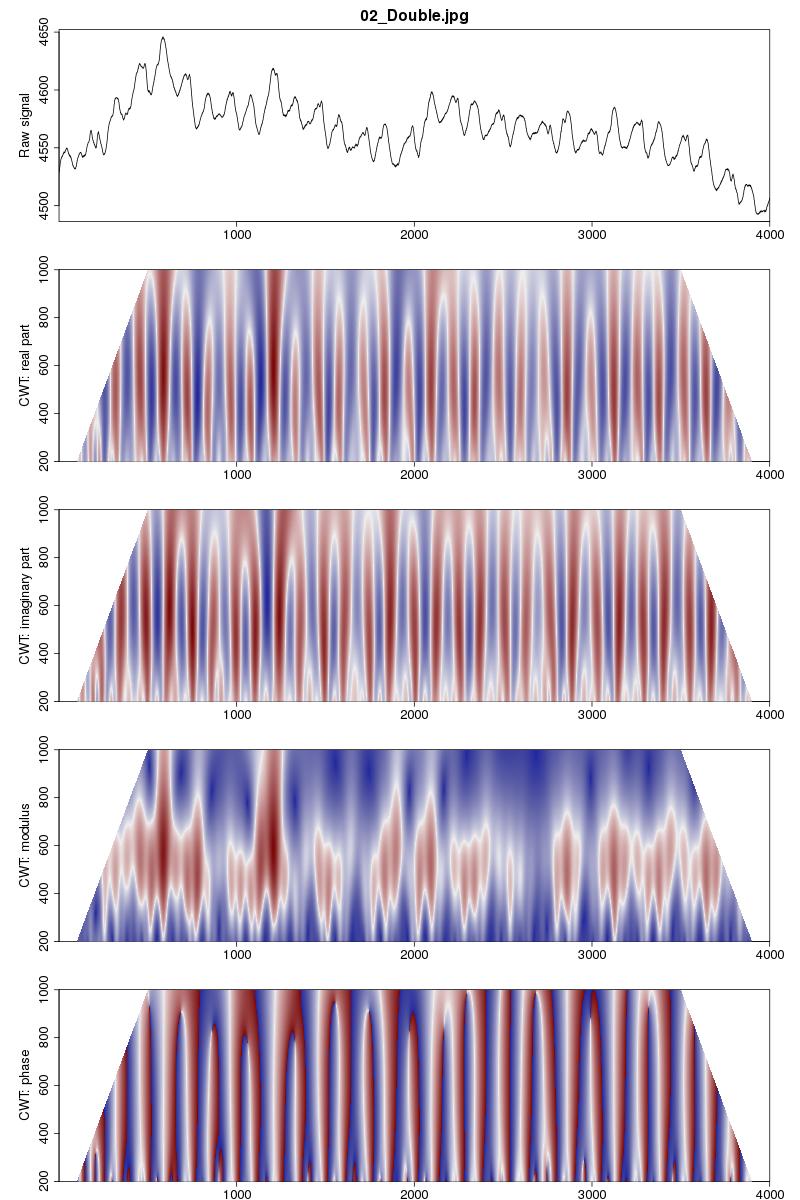

Supplement: S1 Dataset — (XZ) [file pone.0124721.s004.xz › 02_Double.jpg]

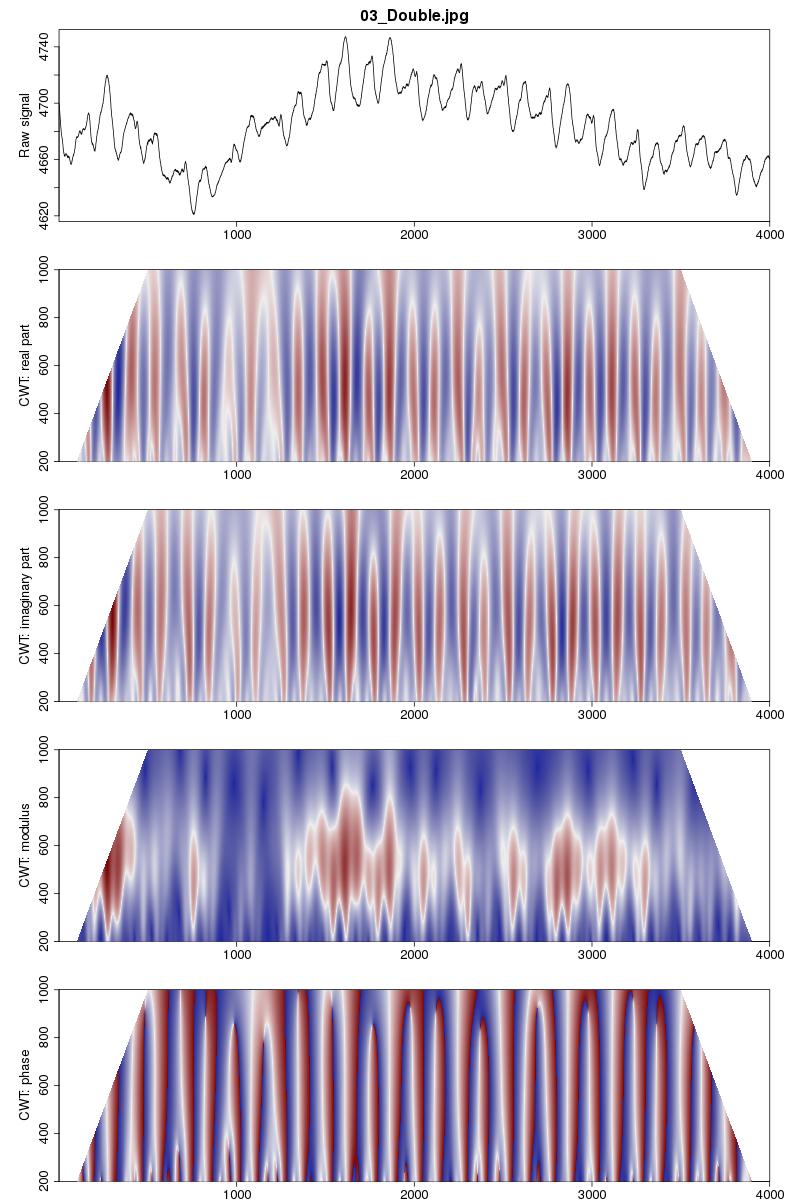

Supplement: S1 Dataset — (XZ) [file pone.0124721.s004.xz › 03_Double.jpg]

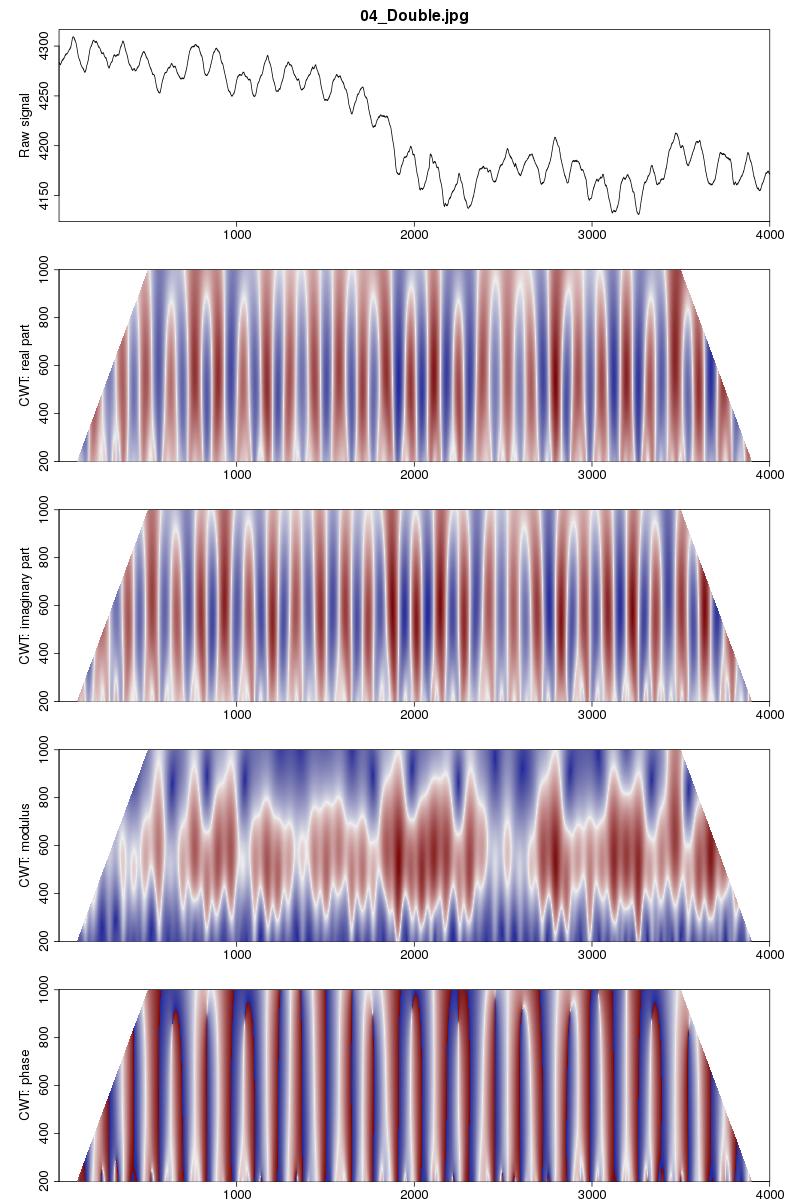

Supplement: S1 Dataset — (XZ) [file pone.0124721.s004.xz › 04_Double.jpg]

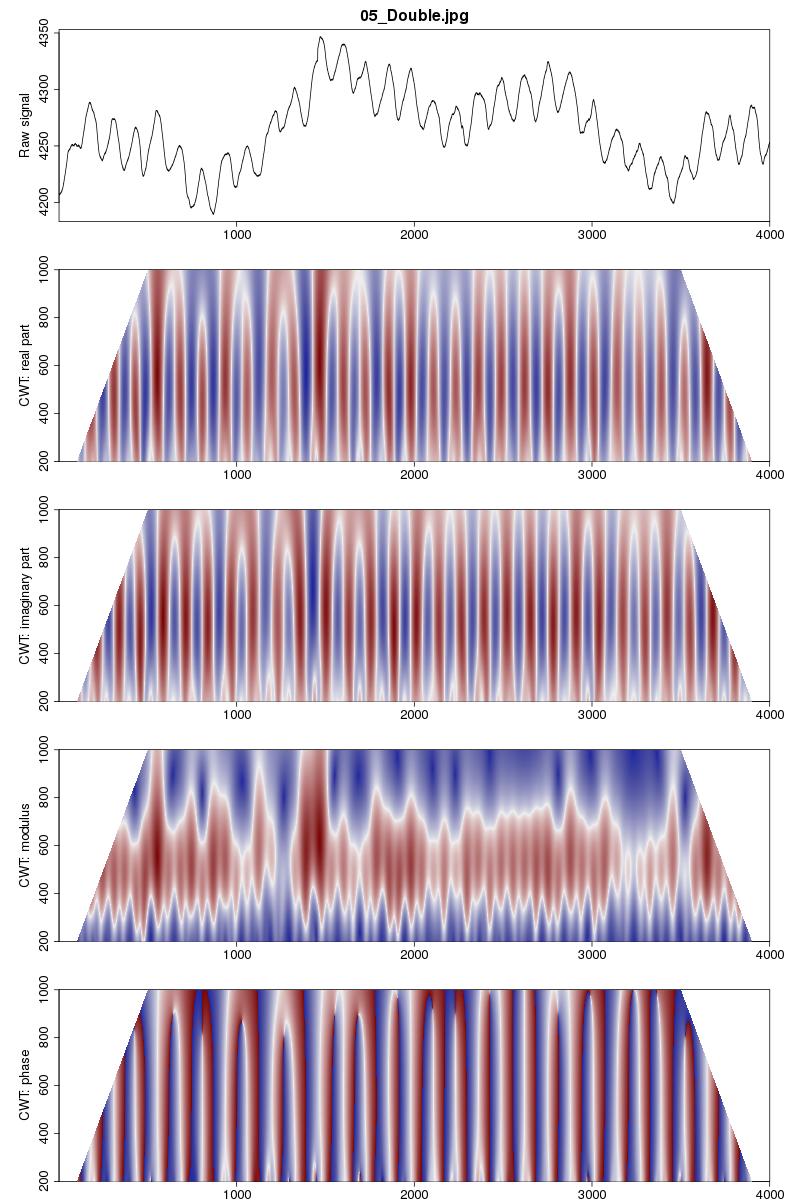

Supplement: S1 Dataset — (XZ) [file pone.0124721.s004.xz › 05_Double.jpg]

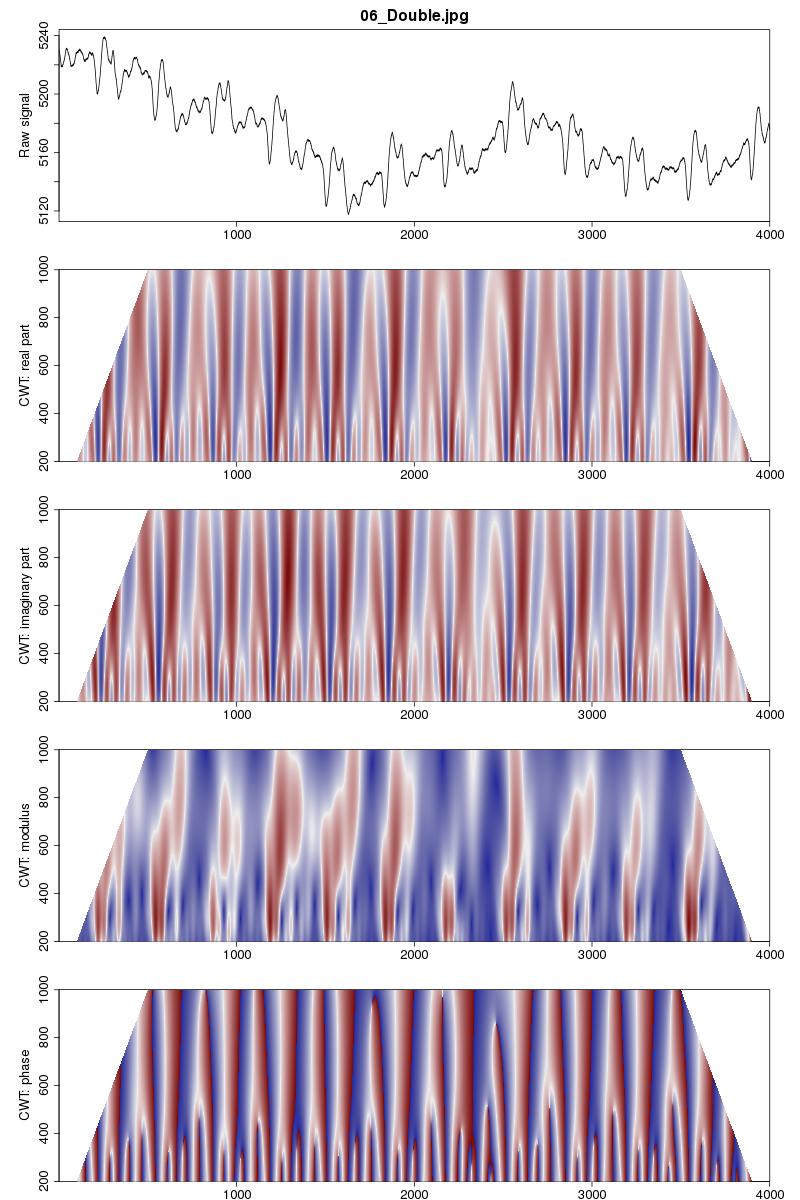

Supplement: S1 Dataset — (XZ) [file pone.0124721.s004.xz › 06_Double.jpg]

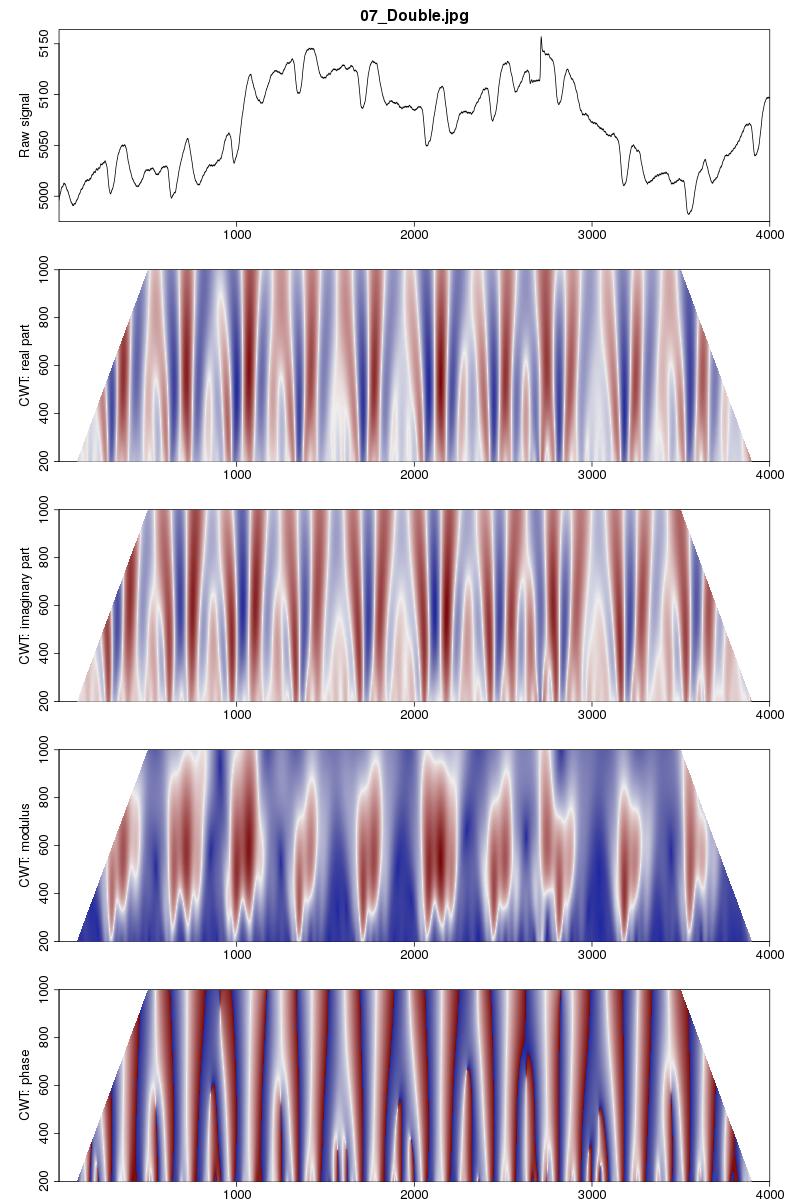

Supplement: S1 Dataset — (XZ) [file pone.0124721.s004.xz › 07_Double.jpg]

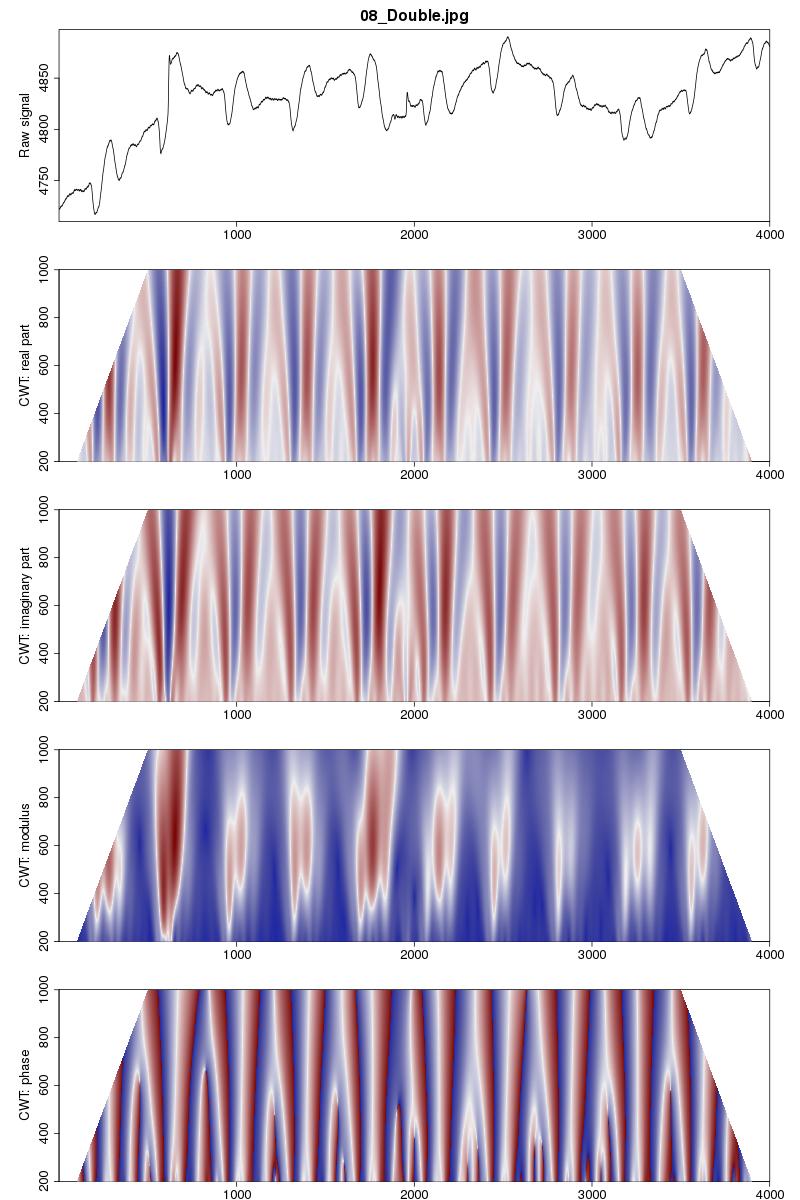

Supplement: S1 Dataset — (XZ) [file pone.0124721.s004.xz › 08_Double.jpg]

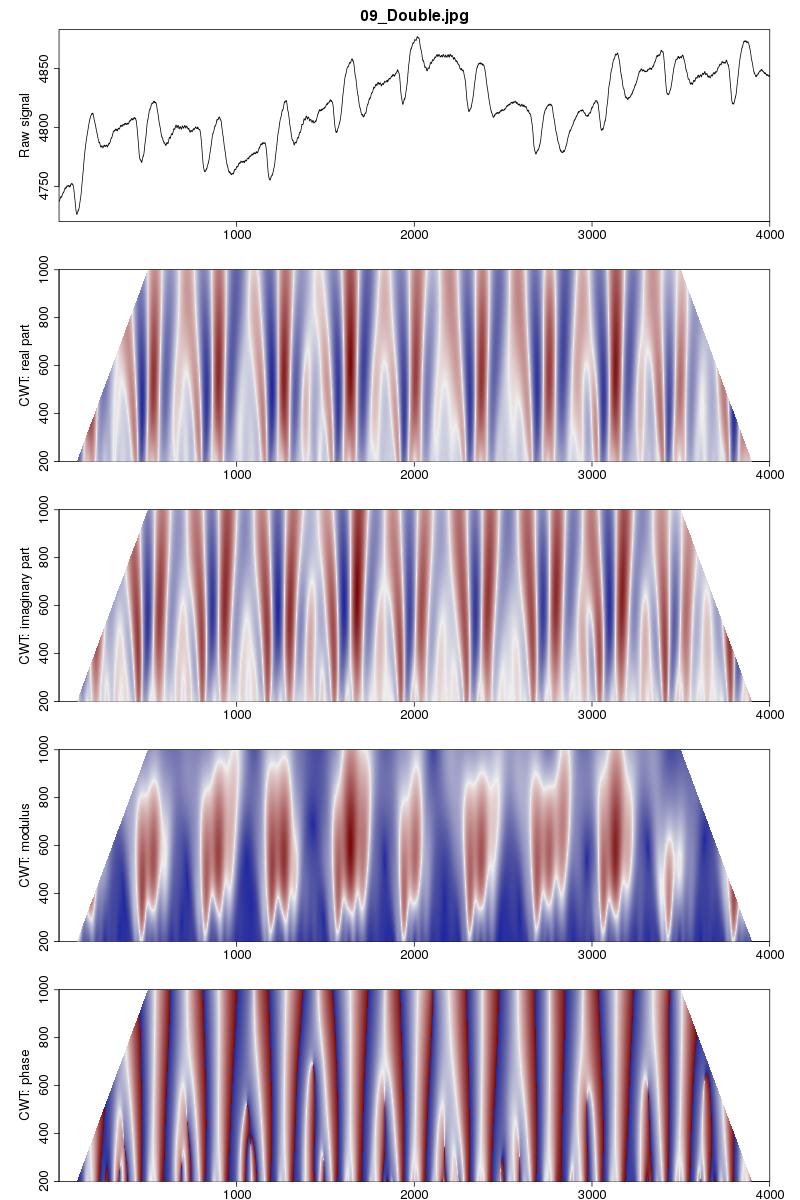

Supplement: S1 Dataset — (XZ) [file pone.0124721.s004.xz › 09_Double.jpg]

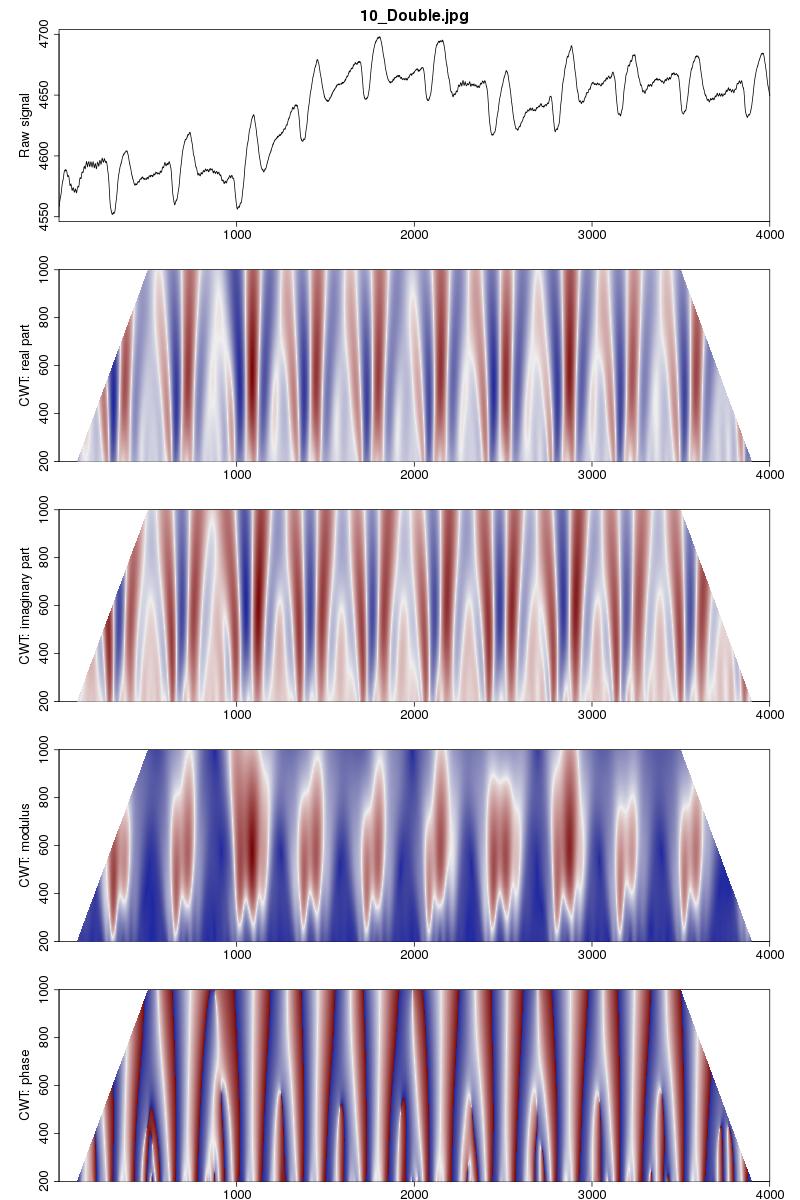

Supplement: S1 Dataset — (XZ) [file pone.0124721.s004.xz › 10_Double.jpg]

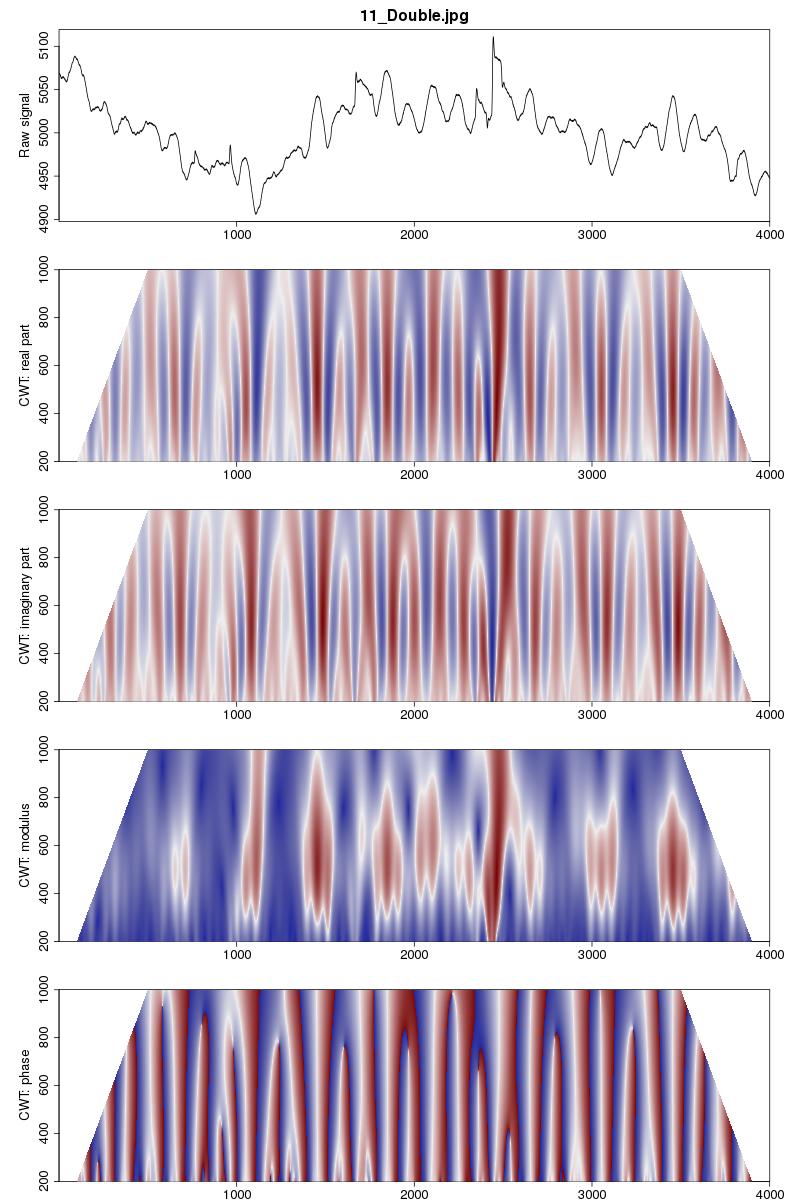

Supplement: S1 Dataset — (XZ) [file pone.0124721.s004.xz › 11_Double.jpg]

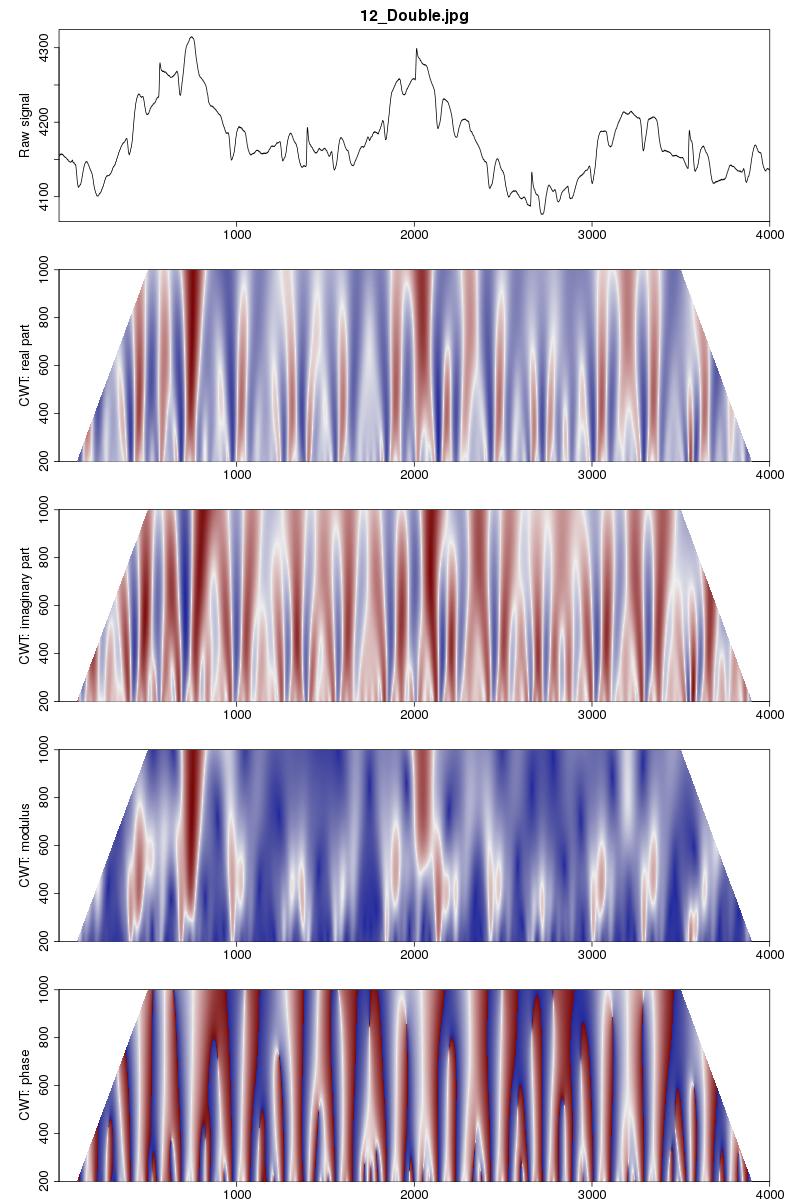

Supplement: S1 Dataset — (XZ) [file pone.0124721.s004.xz › 12_Double.jpg]

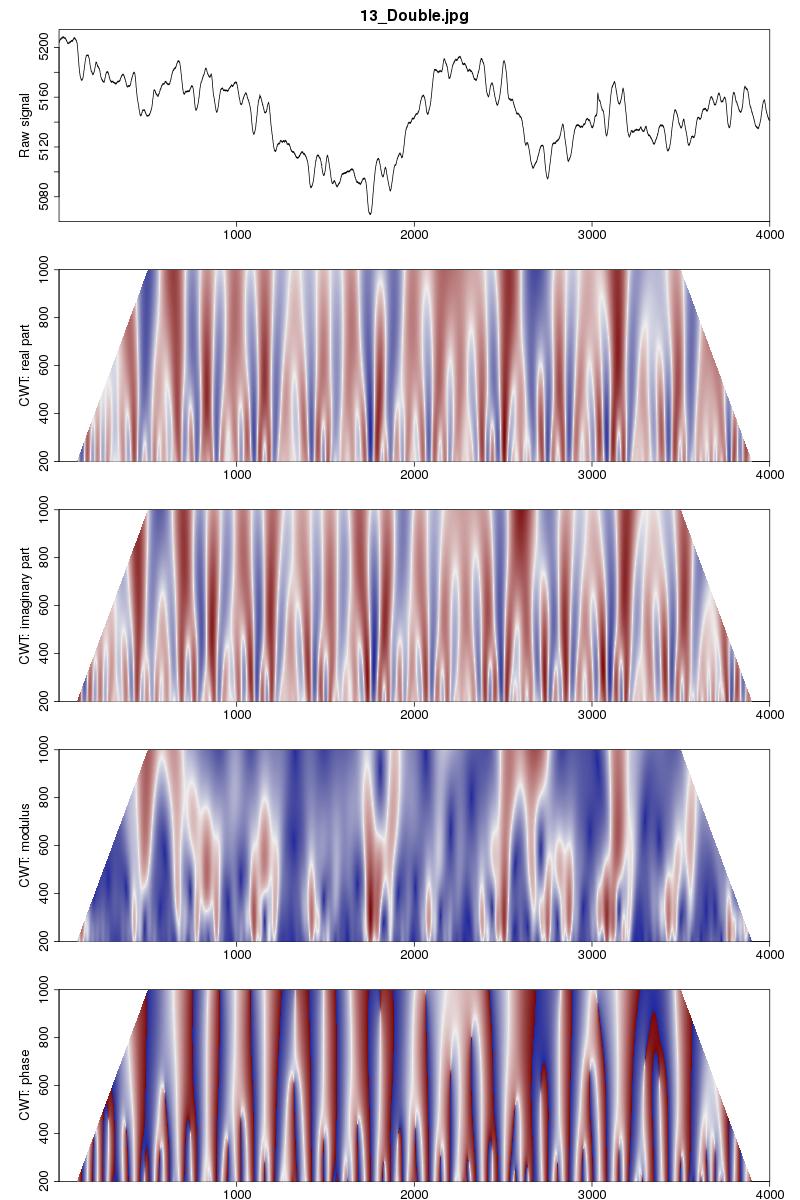

Supplement: S1 Dataset — (XZ) [file pone.0124721.s004.xz › 13_Double.jpg]

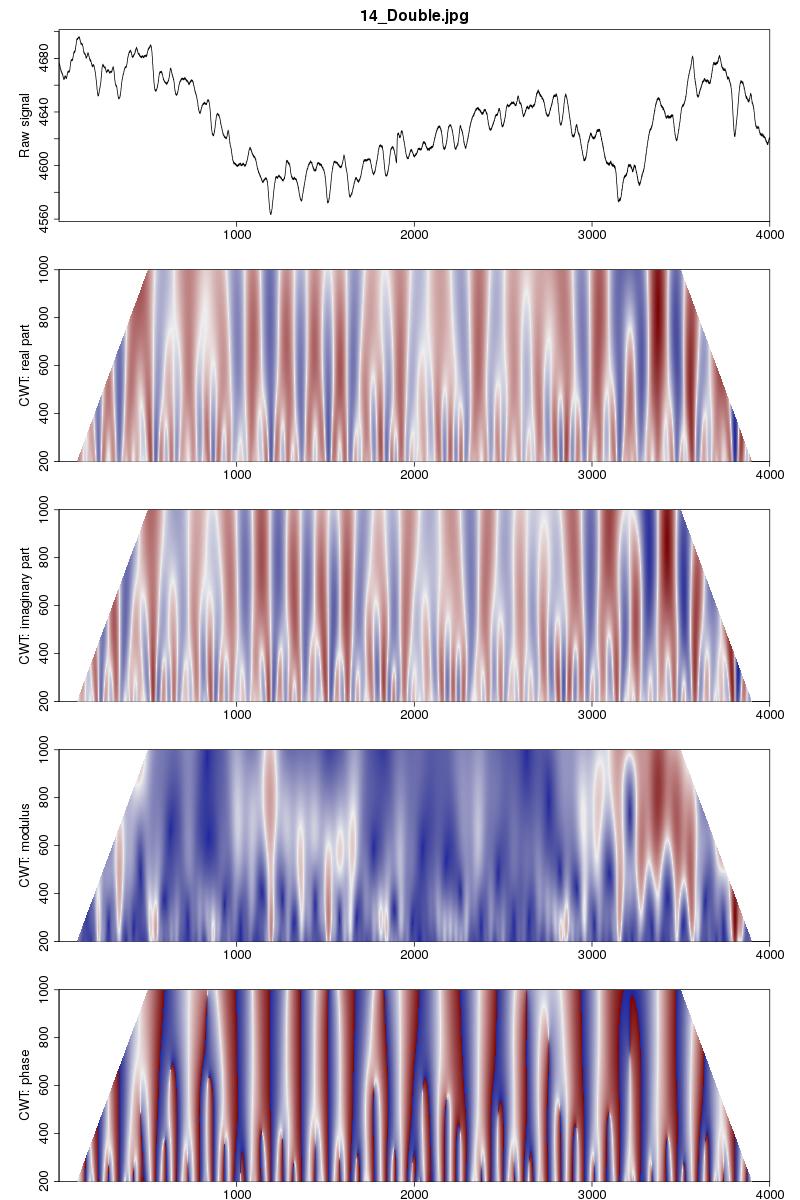

Supplement: S1 Dataset — (XZ) [file pone.0124721.s004.xz › 14_Double.jpg]

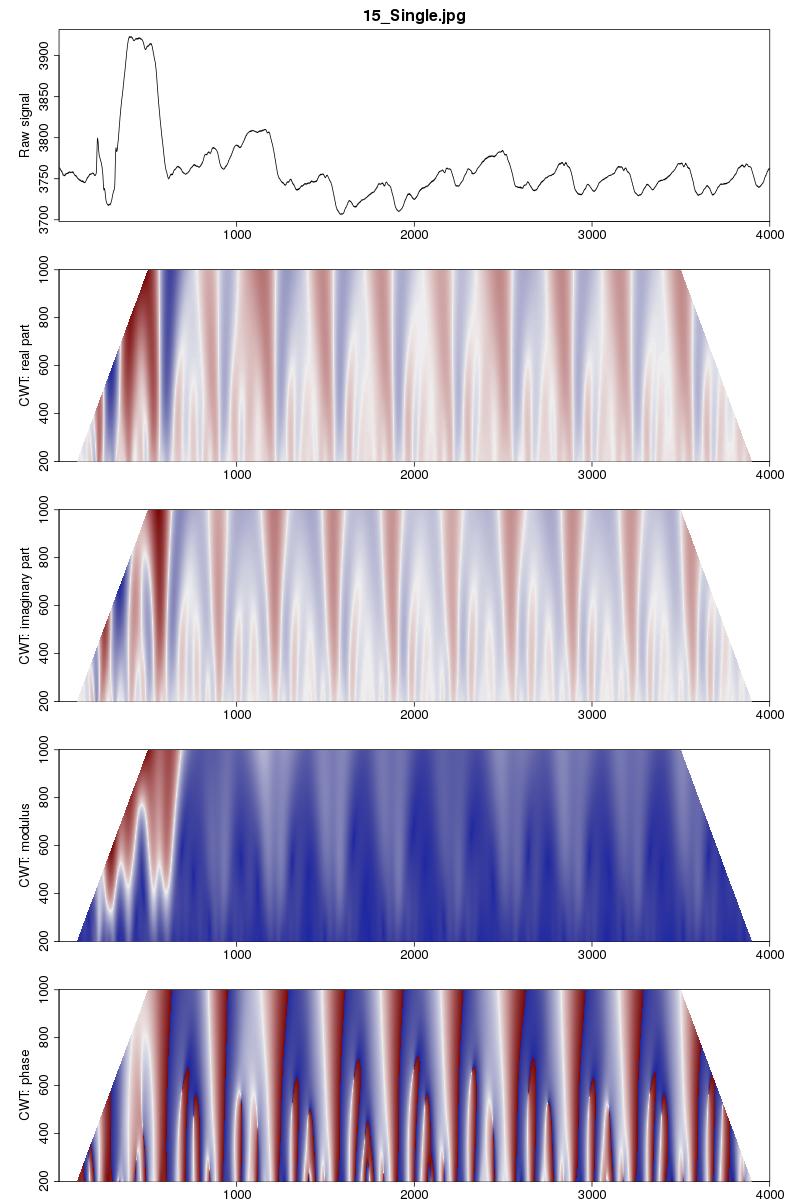

Supplement: S1 Dataset — (XZ) [file pone.0124721.s004.xz › 15_Single.jpg]

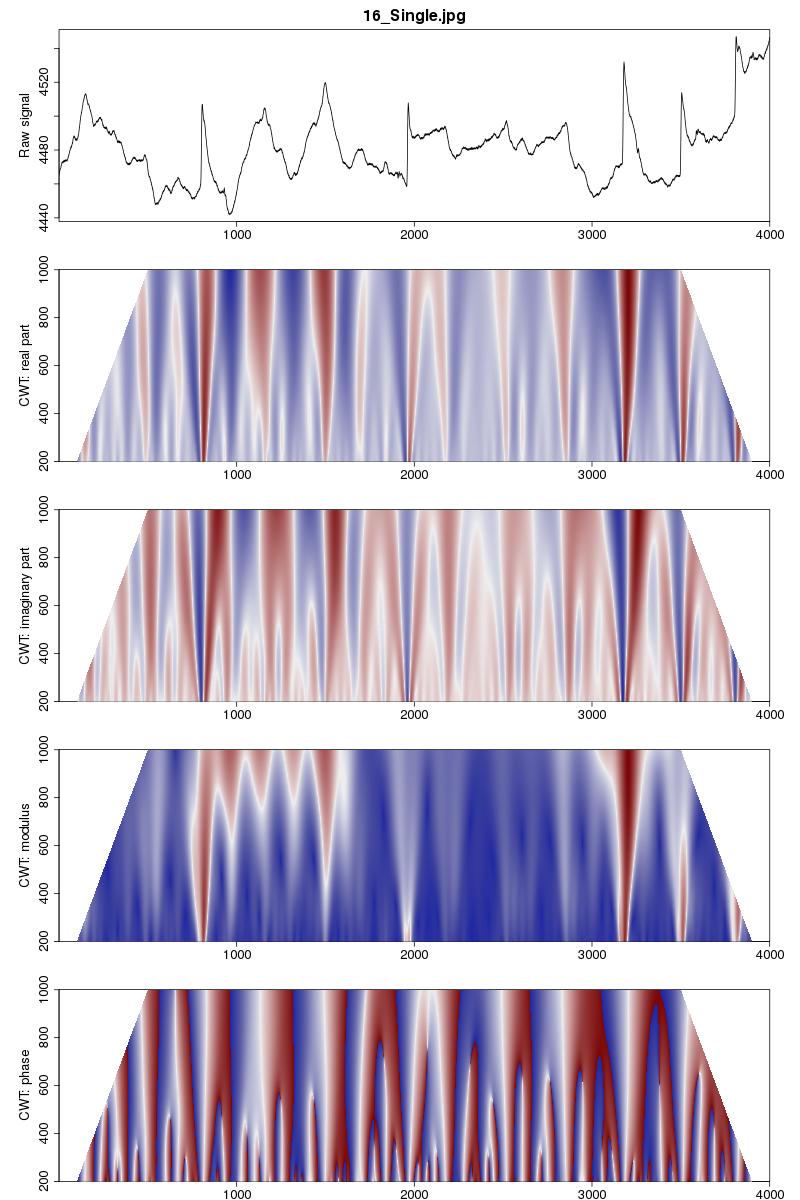

Supplement: S1 Dataset — (XZ) [file pone.0124721.s004.xz › 16_Single.jpg]

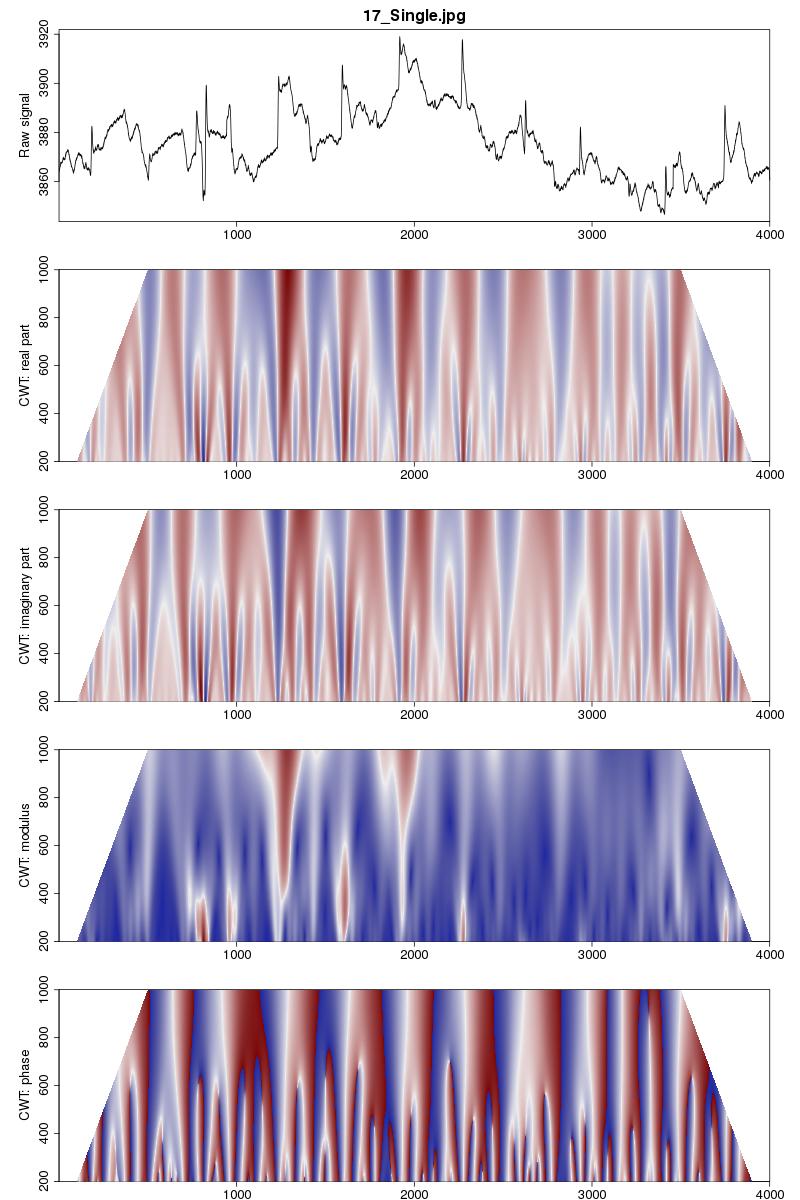

Supplement: S1 Dataset — (XZ) [file pone.0124721.s004.xz › 17_Single.jpg]

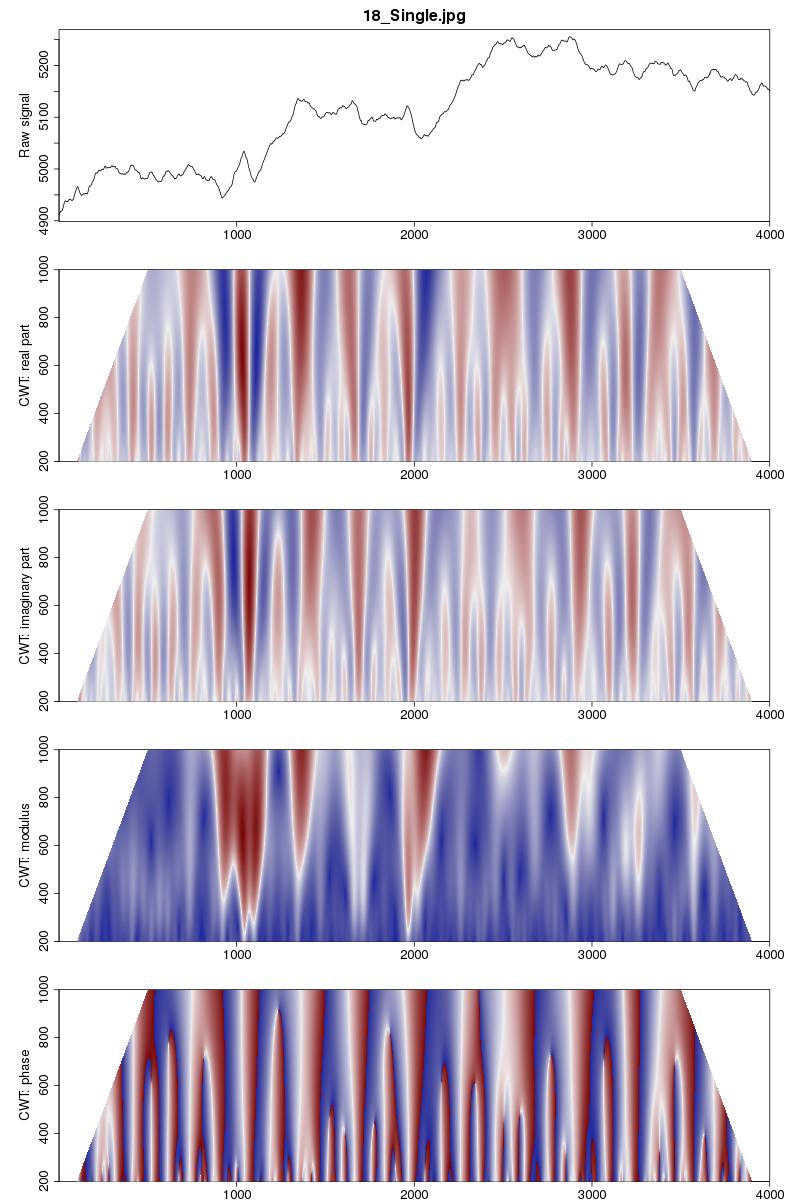

Supplement: S1 Dataset — (XZ) [file pone.0124721.s004.xz › 18_Single.jpg]

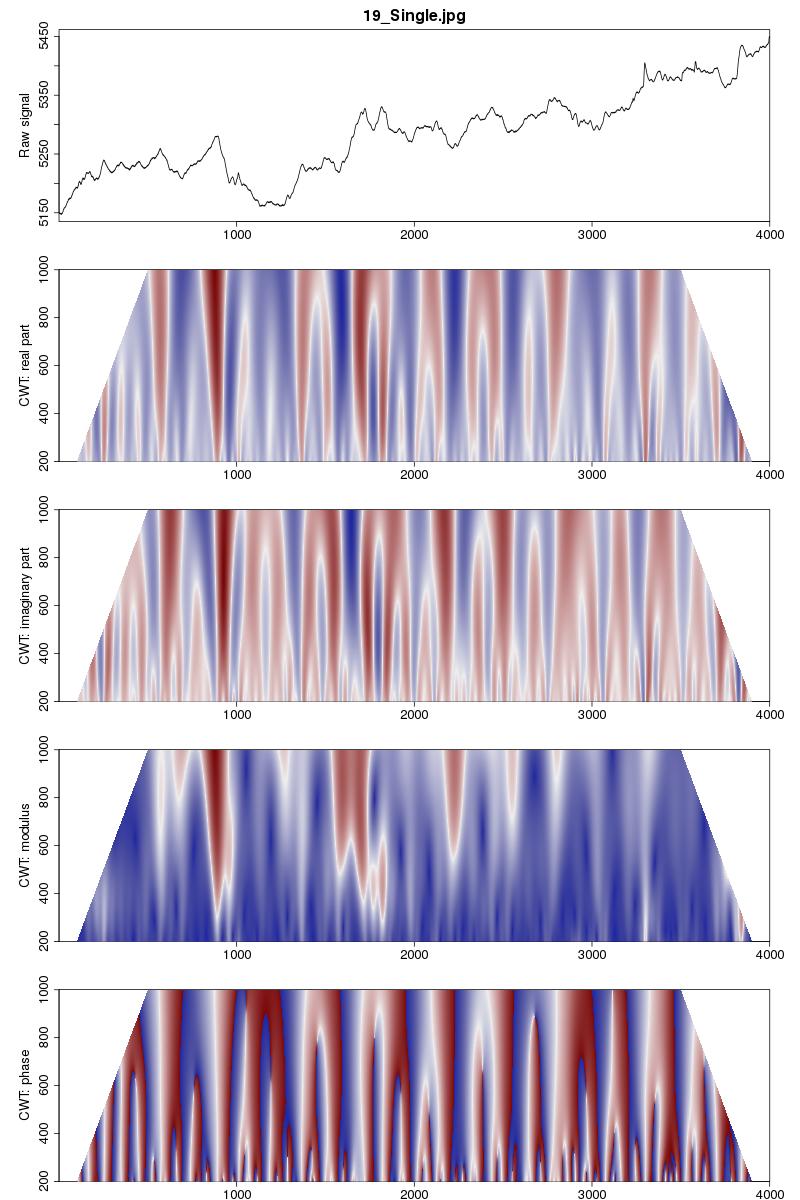

Supplement: S1 Dataset — (XZ) [file pone.0124721.s004.xz › 19_Single.jpg]

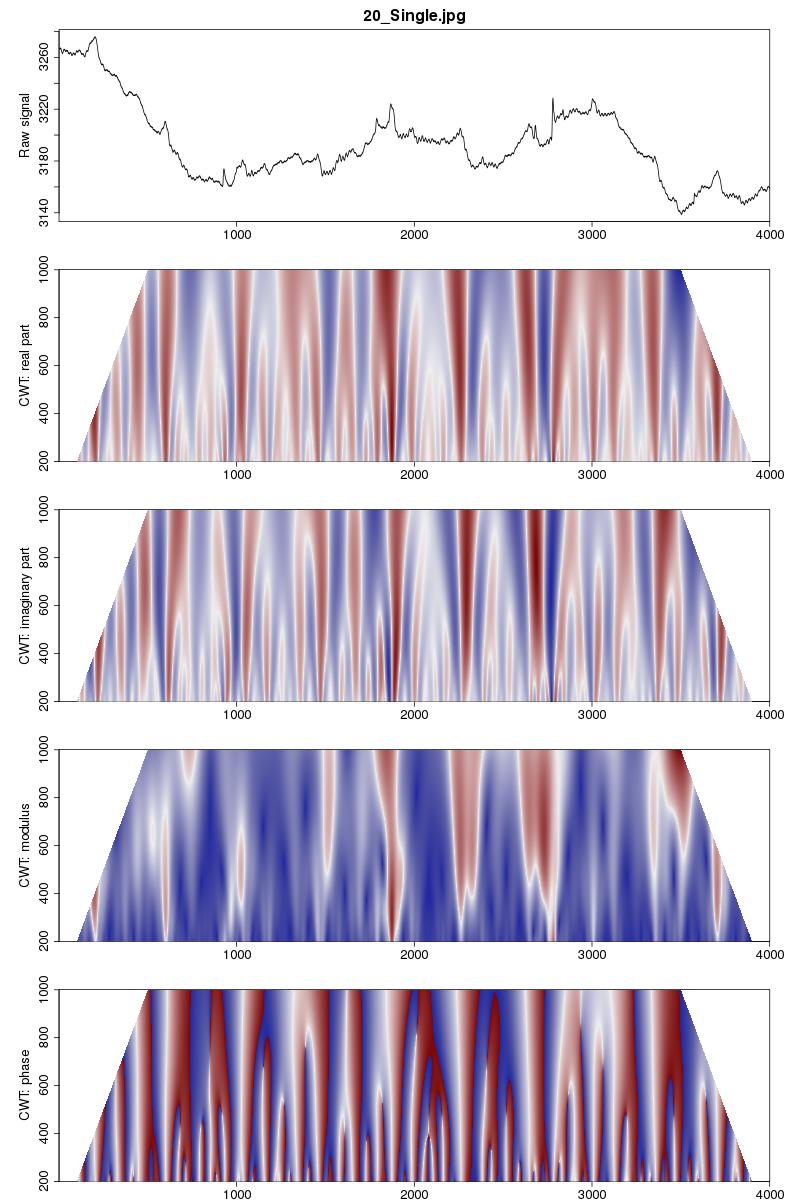

Supplement: S1 Dataset — (XZ) [file pone.0124721.s004.xz › 20_Single.jpg]

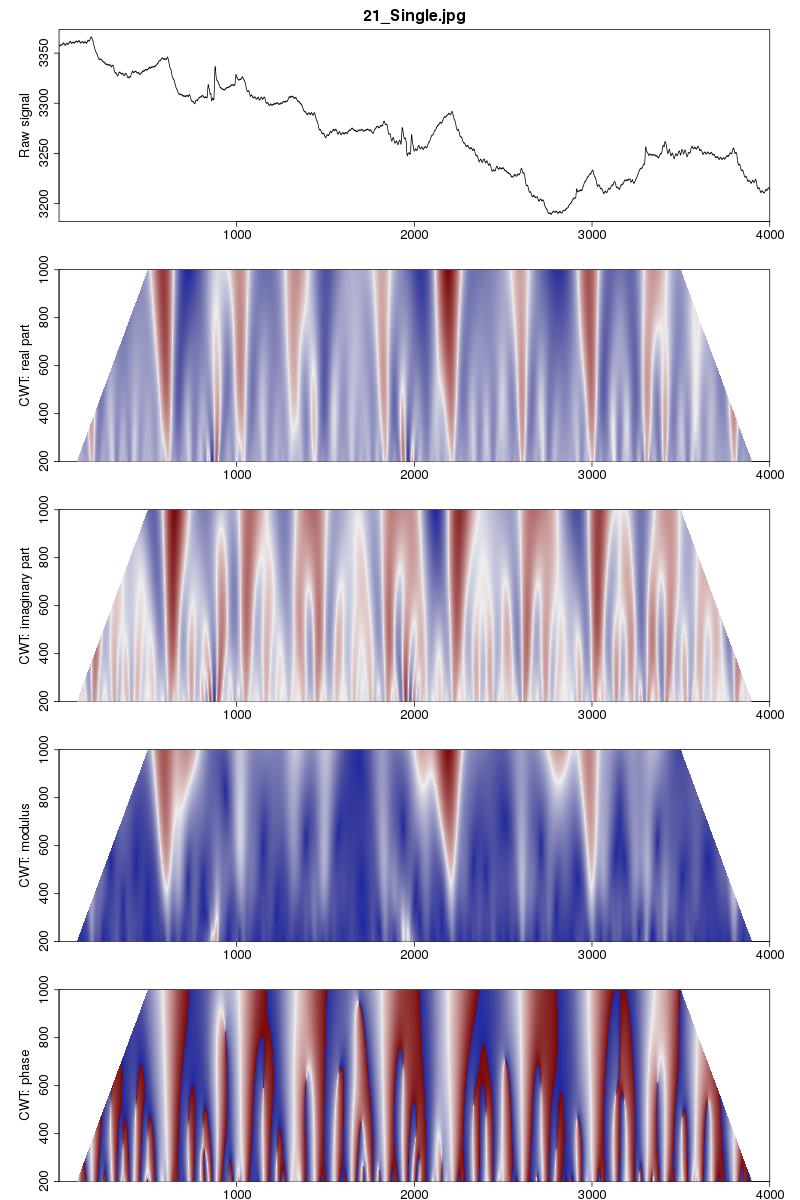

Supplement: S1 Dataset — (XZ) [file pone.0124721.s004.xz › 21_Single.jpg]

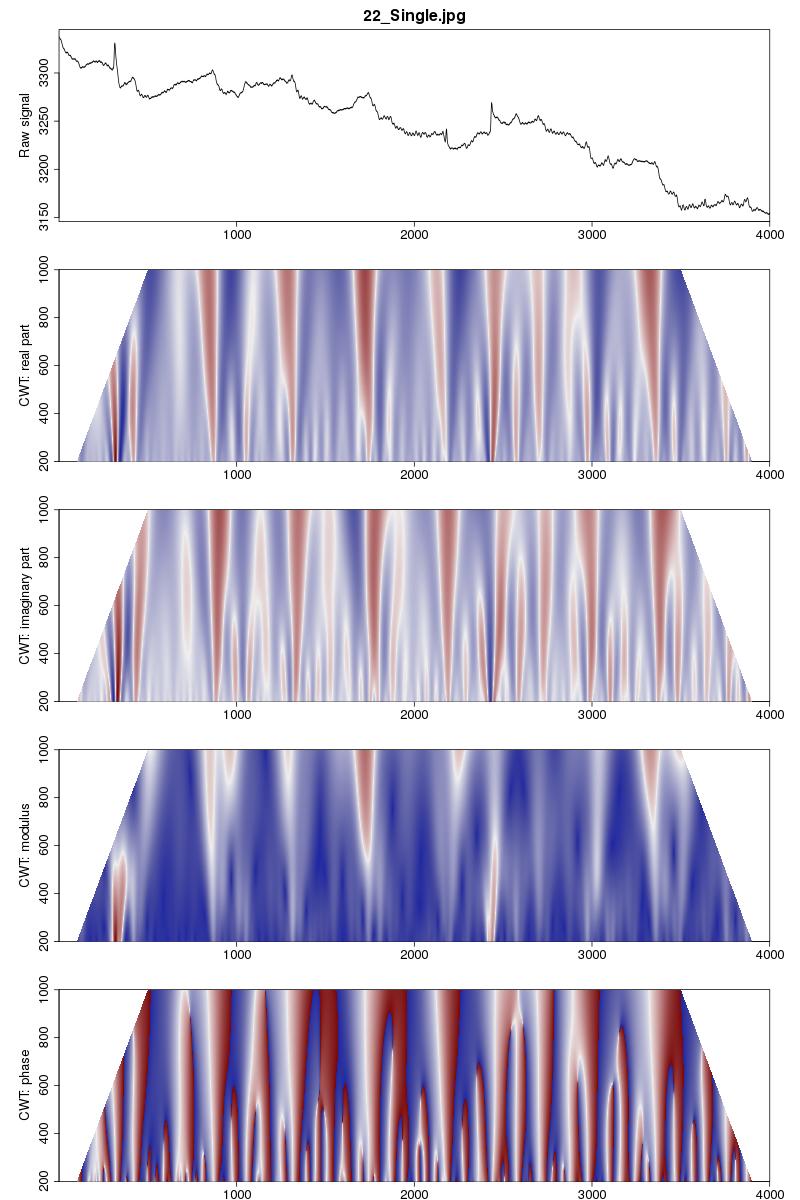

Supplement: S1 Dataset — (XZ) [file pone.0124721.s004.xz › 22_Single.jpg]

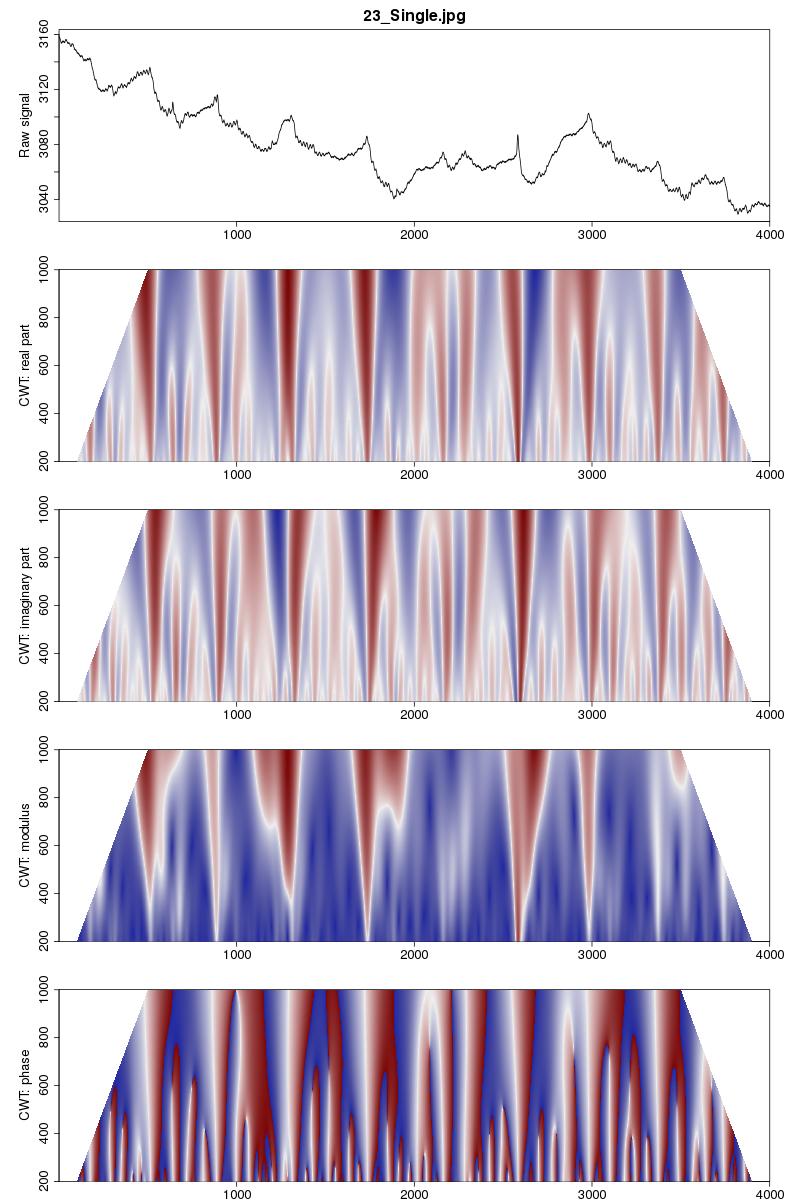

Supplement: S1 Dataset — (XZ) [file pone.0124721.s004.xz › 23_Single.jpg]
